# Are AI Neuroimaging Models Ready for Clinical Use? A Systematic Methodological Review

**DOI:** 10.3390/jcm15093441

**Published:** 2026-04-30

**Authors:** Umid Sulaimanov, Nafiye Sanlier, Ariorad Moniri, Behman Demir, Yerkebulan Serikkanov, Ahmed Rasim Bayramoglu, Maryam Sabah Al-Jebur, Irem Uslu, Oyku Ozturk, Mariagrazia Nizzola, Erkin Ötleş, Simon Gashaw Ammanuel, Abdullah Keles, Ufuk Erginoglu, Mustafa K. Baskaya

**Affiliations:** 1 Department of Neurological Surgery, School of Medicine and Public Health, University of Wisconsin-Madison, Madison, WI 53792, USA; 2 School of Medicine, Acibadem University, Istanbul 34752, Turkey; 3 BerbeeWalsh Department of Emergency Medicine, School of Medicine and Public Health, University of Wisconsin-Madison, Madison, WI 53792, USA

**Keywords:** artificial intelligence, calibration, CLAIM, clinical translation, data leakage, deep learning, external validation, machine learning, medical imaging, TRIPOD-AI

## Abstract

**Background/Objectives**: Artificial intelligence (AI) has rapidly expanded across medical imaging with proposed applications in diagnosis, prognostication, and surgical planning. Concerns remain regarding methodological robustness and clinical readiness for many published models. This systematic review aimed to conduct a methodological audit of AI imaging studies relevant to contemporary neurosurgical practice—including intracranial, cerebrovascular, spinal, and connectomics-based applications—published in 2025. **Methods**: Following PRISMA guidelines and PROSPERO registration (CRD420261284068), PubMed was searched for studies published in 2025 evaluating machine learning or deep learning applications in MRI- or CT-based imaging. Three reviewers independently extracted data on validation strategy, data leakage risk, human comparator use, calibration reporting, and CLAIM/TRIPOD-AI adherence. Risk of bias was assessed using PROBAST+AI. **Results**: Of 1776 screened records, 91 studies met the inclusion criteria. China led contributions (54.9%), oncology was the most common domain (37.4%), and MRI was the predominant modality (67.0%). External validation was reported in 75.8% of studies, and 66.0% used multicenter cohorts. Data leakage risk was low in 93.4%. However, only 18.7% included human comparators, calibration was reported in 30.8%, and none achieved full CLAIM/TRIPOD-AI compliance. **Conclusions**: AI imaging studies published in 2025 demonstrate encouraging progress in multicenter design and external validation. However, persistent gaps in human benchmarking, calibration, and reporting suggest further methodological development is needed.

## 1. Introduction

Artificial intelligence (AI) technologies have gained considerable traction in medical imaging research. Machine learning (ML) and deep learning (DL) models are increasingly proposed for predictive purposes, including prognostication, diagnosis, and surgical planning. AI applications have become especially widespread in oncologic imaging but also demonstrated their relevance in neurologic and neuro-radiologic diagnostics, surgery, and other fields. The performance of AI systems in various clinically valuable tasks is quite promising, including tumor characterization, hemorrhage detection, vascular risk estimation, and outcome modeling. Thus, an increased number of AI-based studies are now being published in radiology and associated disciplines.

Alongside this proliferation, there appear to be numerous problems related to methodological robustness and generalizability of many models being used. The adoption of models not validated well enough may result in serious issues, as seen by the example of clinically used AI systems, which revealed themselves to be much less efficient than initially claimed [1,2]. Previously published systematic reviews outlined major problems associated with insufficient external validation, poor generalizability, methodological biases, potential data leakage, and insufficiently transparent reporting [3,4,5]. In addition, many studies still do not fully conform to relevant guidelines for reporting (particularly Checklist for Artificial Intelligence in Medical Imaging (CLAIM) and Transparent Reporting of a multivariable prediction model for Individual Prognosis or Diagnosis using Artificial Intelligence (TRIPOD-AI)) despite the increasing awareness of the problem. Finally, the role of Consolidated Standards of Reporting Trials for Artificial Intelligence (CONSORT-AI) as a supplement to clinical trials evaluating AI should not be underestimated [6,7,8]. Furthermore, strong discriminatory performance alone does not ensure clinical utility. Adequate calibration, seamless workflow integration, and robust inter-institutional transferability are often lacking.

To address some of these problems, several checklists such as CLAIM, TRIPOD-AI, and CONSORT-AI were developed in order to promote more transparent and robust reporting of AI studies [6,7,8]. Despite the increasing recognition of these frameworks, however, many recently published evaluations concentrated on certain aspects or specific checklists instead of conducting a more thorough analysis of validation strategies, data leakage risk, calibration, clinical benchmarking, and reporting compliance.

In light of all this, the present study sought to conduct a comprehensive methodological assessment of studies employing AI in surgical practice published in 2025. To our knowledge, no prior systematic review has provided a contemporaneous, year-specific methodological audit of validation methods, risk of data leakage, calibration, benchmarking against human evaluators, and reporting quality within this field. Instead of making comparisons in terms of discriminatory power (AUC, etc.), we conducted a systematic assessment of datasets, validation procedures, AI models employed, comparison to human experts, calibration, and compliance with reporting standards.

## 2. Materials and Methods

The research protocol for this systematic review was registered in the International Prospective Register of Systematic Reviews (PROSPERO ID: CRD420261284068).

### 2.1. Study Identification

The PubMed database was systematically searched in accordance with the Preferred Reporting Items for Systematic Reviews and Meta-Analyses (PRISMA) guidelines [9]. Eligible studies were those presenting primary data on AI applications in medical imaging involving human subjects.

The following Boolean search strategy was employed: (“Artificial Intelligence” OR “Machine Learning” OR “Neural Networks, Computer” OR “artificial intelligence” OR “machine learning” OR “deep learning” OR “neural network*” OR “radiomics”) AND (“Diagnostic Imaging” OR “medical imaging” OR “radiology” OR “MRI” OR “CT”) AND (“bias” OR “external validation” OR “generalizability” OR “reproducibility” OR “data leakage”) AND (“Humans”) AND (“2025/01/01” [Date–Publication]: “2025/12/31” [Date–Publication]).

### 2.2. Eligibility Criteria

Articles were considered eligible for inclusion if they discussed the use of AI techniques in medical imaging relevant to neurosurgical practice and fulfilled the following criteria: (1) publication in 2025; (2) original research article; (3) application of AI methods on MRI and CT imaging modalities and/or angiographic imaging (i.e., computed tomography angiography (CTA) and magnetic resonance angiography (MRA)); (4) human subjects’ studies; (5) development, validation, and/or evaluation of models; and (6) availability of the full texts. For the purpose of this review, “relevant to neurosurgical practice” was defined to include: (a) intracranial pathology and neurological disease (e.g., tumors, stroke, hemorrhage, epilepsy, hydrocephalus, aneurysms); (b) spinal pathology routinely managed by neurosurgeons (e.g., degenerative spine disease, vertebral fractures, surgical planning and screw placement); (c) extracranial cerebrovascular disease contributing to stroke or requiring surgical or endovascular intervention; and (d) connectomics and functional imaging studies with direct applicability to neurosurgical planning workflows (e.g., tractography, eloquent-area mapping, DBS targeting). Studies originating from related clinical specialties (orthopedics, otolaryngology, radiology, neuroscience) were considered eligible when their investigated imaging conditions fell within one of these categories.

Papers that did not meet the following criteria were excluded: (1) review papers, systematic reviews, and/or meta-analysis; (2) conference abstracts, editorials, letter to editors, or comments; (3) studies involving only animals, phantoms, or simulations; (4) purely technical articles in computer sciences with no medical imaging applications; (5) AI articles irrelevant to medical imaging; (6) papers not applying machine learning/deep learning algorithms; and (7) papers published before 2025.

### 2.3. Study Selection Process

**Figure 1:**
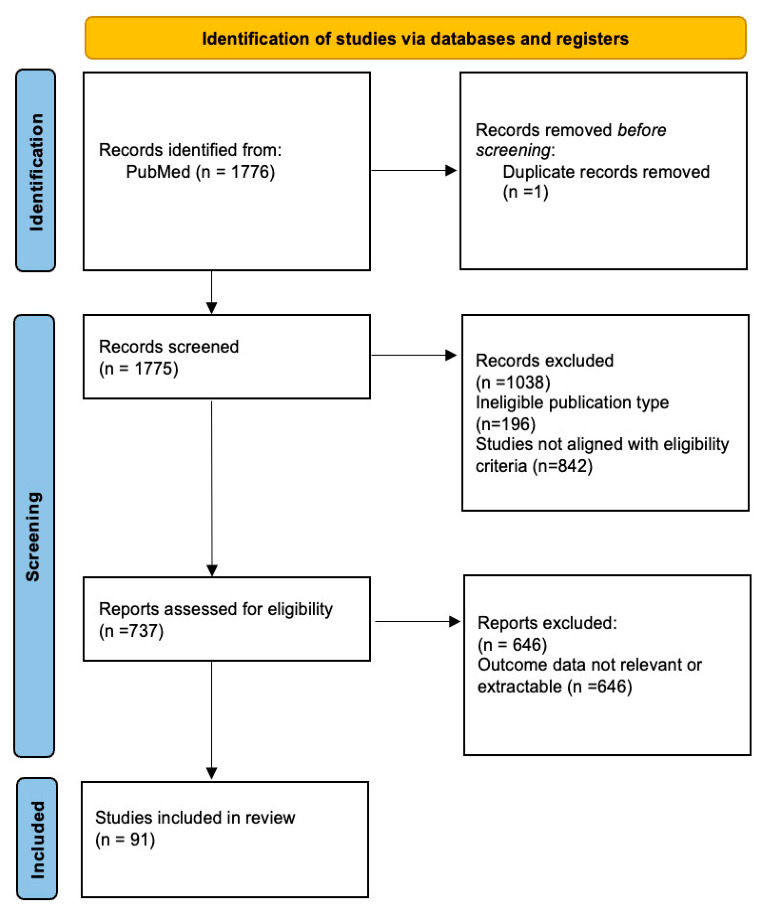
PRISMA flow diagram illustrating the study selection process.

Duplicated entries were eliminated via Rayyan. Titles and abstracts were then independently reviewed by three reviewers (U.S., N.S., and B.D.) based on their eligibility for further consideration. The full texts of articles identified as eligible by the aforementioned three independent reviewers were subsequently evaluated again for the purpose of finalizing the article selections for this systematic review. Any disagreements between the independent reviewers were settled through discussion and consensus among all reviewers. At each step of the selection procedure, reviewers reached agreement via consensus building. Reference lists of the selected papers were manually checked for any additional eligible citations. The study selection process followed the PRISMA guidelines, and the selection flowchart can be found in Figure 1.

### 2.4. Data Extraction

Data were independently collected by three reviewers using a standardized extraction form, with any disagreements resolved through discussion and consensus. For each included study, information was gathered across several domains: (1) study characteristics (author, publication year, journal, country, and medical field); (2) imaging and dataset features (imaging modality, dataset type, sample size, and ground truth definition); (3) AI model details (type of AI, model architecture, and task). Hybrid models were defined as those combining deep learning and traditional machine learning within a single pipeline. In multi-component pipelines, classification was based on the primary modeling approach described. Additional domains were (4) validation and transparency (validation method, use of external datasets, clarity of data splitting, and risk of data leakage); (5) methodological features (study design, inclusion of human comparators, reporting of calibration metrics, clarity of performance metrics, and adherence to CLAIM/TRIPOD-AI guidelines); and (6) reported outcomes (main performance metric and claims of clinical applicability). Studies involving multiple clinical domains were categorized as mixed.

Variables were recorded as either categorical or numerical, as appropriate (e.g., sample size as numerical; split clarity, external validation, human comparator, calibration reporting, and clinical applicability as yes/no; and data leakage risk and CLAIM/TRIPOD-AI adherence as predefined categories). The extracted data were summarized using descriptive statistics.

Data leakage risk was evaluated based on reported data handling and validation procedures, guided by CLAIM and TRIPOD-AI principles. Risk was classified as low when training, validation, and test sets were clearly separated at the patient or center level without overlap. Moderate risk existed when the data division was vague or described in an equivocal manner. High or unclear risk was characterized by clear overlap between datasets, preprocessing steps undertaken prior to data splitting, or inadequate reporting to eliminate leakage. The clarity of split descriptions was marked as “yes” when studies clearly detailed how datasets were divided (including whether splitting was done at the patient or center level) and “no” when this information was incomplete or unclear.

Adherence to CLAIM and TRIPOD-AI was evaluated at the domain level rather than through formal item-level scoring. Six predefined reporting domains derived from both frameworks were assessed: (1) data partitioning transparency; (2) model development and architecture clarity; (3) validation methodology; (4) completeness of performance reporting; (5) calibration reporting; and (6) human comparator analysis, when applicable. Three reviewers (U.S., N.S., B.D.) assessed each study independently and reconciled by consensus. Studies were classified as showing full adherence (all applicable domains adequately reported), partial adherence (one or more domains incompletely reported), or no adherence (key domains absent). Full item-level scoring (42 CLAIM items, 27+ TRIPOD-AI items) across 91 heterogeneous studies was beyond the feasible scope of this field-level audit. Direct comparisons with expert performance by radiologists or neuroradiologists on the same data and task were recorded as “yes” for human comparators; research lacking such comparisons was categorized as “no”. Calibration reporting was considered present if any standard metric (e.g., calibration curves, Brier score, Hosmer–Lemeshow test, or equivalent) was reported.

### 2.5. Data Analysis

The analysis of the data was conducted as a descriptive systematic review. Counts and proportions were used to summarize extracted variables. The multinational studies were those studies that were performed in two or more countries. In the case of multiple imaging modalities, the primary modality was used to classify them. The variables that were categorical (e.g., the type of dataset, the validation strategy, external validation, data leakage risk, calibration reporting, and the use of human comparators) were reported as frequencies and percentages.

Descriptive measures (means or medians, depending on the type of continuous variables) were used to summarize continuous variables (sample size, performance metrics, e.g., AUC, accuracy). Because of the high level of heterogeneity in study designs, protocols of imaging, patient groups, measures of outcomes, and reporting of outcomes, a meta-analysis was not performed.

Subgroup analyses were done where they were necessary, especially studies with or without external validation. Synthesized findings were presented in narrative form, and the quality of the methods, practices of validation, and possible biases in relation to clinical use were considered.

### 2.6. Risk of Bias Assessment

The risk of bias was assessed by three reviewers (U.S., N.S., and B.D.) with the help of Prediction Model Risk of Bias Assessment Tool of AI (PROBAST+AI) [10]. All studies were evaluated on the following areas: participants, predictors, outcomes, and analysis, where the risk of bias was evaluated, in general, according to the guidelines of PROBAST+AI.

## 3. Results

### 3.1. Study Selection

The initial search in the database revealed 1776 records. After removing 1 duplicate, 1775 records were screened by title and abstract. Subsequently, 737 full-text articles were assessed for the final screen. Finally, 91 studies were eligible for the inclusion criteria and were used in the qualitative synthesis. Figure 1 (PRISMA flow diagram) presents a description of the selection procedure.

### 3.2. General Study Characteristics

**Figure 2:**
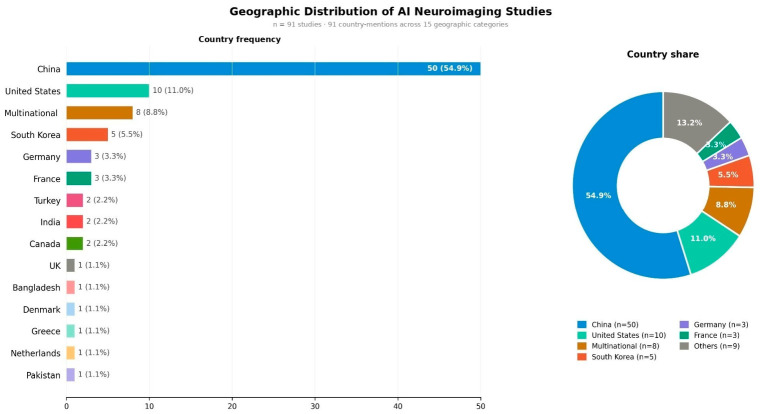
Geographic distribution of included AI neuroimaging studies (*n* = 91). The bar chart (**left**) displays the frequency of studies by country of origin across 15 geographic categories. The donut chart (**right**) illustrates the proportional country share. China was the predominant contributor (*n* = 50, 54.9%), followed by the United States (*n* = 10, 11.0%) and multinational collaborations (*n* = 8, 8.8%).

The 91 studies that were included were very widely internationally represented and represented 14 countries and 8 multinationals. China contributed the largest share (54.9%, *n* = 50), followed by the United States (11.0%, *n* = 10) and multinational studies (8.8%, *n* = 8) (Figure 2).

**Figure 3:**
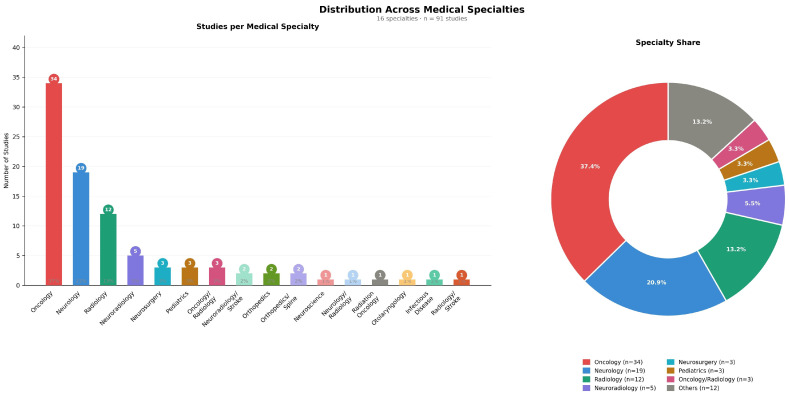
Distribution of included studies across medical specialties (*n* = 91, 16 specialties). The bar chart (**left**) shows the number of studies per specialty. The donut chart (**right**) illustrates the proportional specialty share. Oncology represented the largest group (*n* = 34, 37.4%), followed by neurology (*n* = 19, 20.9%) and radiology (*n* = 12, 13.2%).

The most represented field was oncology (34 studies, 37.4%). This was followed by neurology (*n* = 19, 20.9%), radiology (*n* = 12, 13.2%), and neuroradiology (*n* = 5, 5.5%). It was also found that multidisciplinary studies were done, such as oncology/radiology (*n* = 3, 3.3%) and neurology/radiology (*n* = 1, 1.1%). The proportion of all other specialties was below 5% each (Figure 3).

MRI was the most common imaging modality (61 of 91 studies, 67.0%). The remaining 30 studies (33.0 percent) were founded on CT imaging, such as non-contrast CT, CT angiography, and similar methods.

### 3.3. Artificial Intelligence Methodological Characteristics

**Figure 4:**
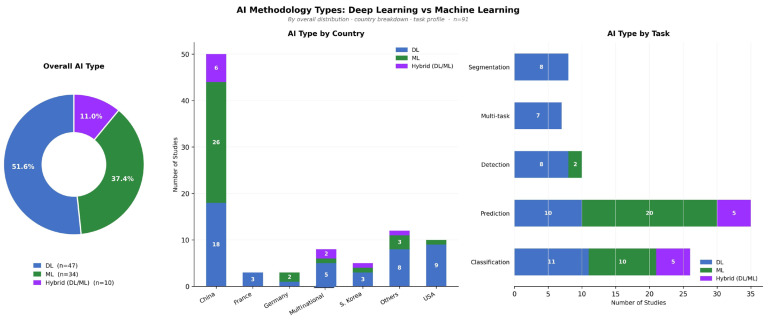
AI methodology types across included studies: deep learning versus machine learning (*n* = 91). The donut chart (**left**) shows the overall distribution of AI types: deep learning (DL, 51.6%), machine learning (ML, 37.4%), and hybrid DL/ML (11.0%). The stacked bar charts illustrate AI type distribution by country (**center**) and by clinical task (**right**). DL, deep learning; ML, machine learning.

The most widespread method was the use of deep learning (DL), which was mentioned in 47 studies (51.6%) [11,12,13,14,15,16,17,18,19,20,21,22,23,24,25,26,27,28,29,30,31,32,33,34,35,36,37,38,39,40,41,42,43,44,45,46,47,48,49,50,51,52,53,54,55,56,57]. Machine learning (ML) methods were used in 34 studies (37.4%) [58,59,60,61,62,63,64,65,66,67,68,69,70,71,72,73,74,75,76,77,78,79,80,81,82,83,84,85,86,87,88,89,90,91], while hybrid DL/ML approaches were reported in 10 studies (11.0%) [92,93,94,95,96,97,98,99,100,101] (Figure 4). In general, DL was observed, but traditional ML was still used extensively. China had the most studies of any category of methodology (Figure 4).

**Figure 5:**
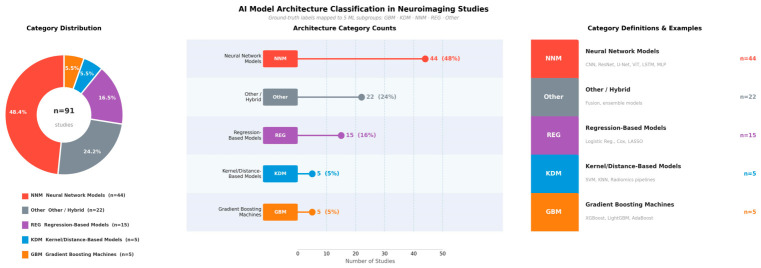
AI model architecture classification in neuroimaging studies (*n* = 91). Ground-truth labels were mapped to five subgroups. The donut chart (**left**) shows the proportional distribution. The lollipop chart (**center**) displays absolute counts and percentages per category. The legend (**right**) provides category definitions and clinical examples. NNM, neural network models; REG, regression-based models; KDM, kernel/distance-based models; GBM, gradient boosting machines.

The most common architecture was the neural network-based models, with 44 studies (48.4%). The next most common models were hybrid or ensemble models (*n* = 22, 24.2%), followed by regression-based models in 15 studies (16.5%). In 5 studies (5.5%), kernel/distance-based methods and gradient boosting were reported. These results demonstrate the supremacy of neural networks, with the ongoing application of traditional and hybrid approaches (Figure 5).

**Figure 6:**
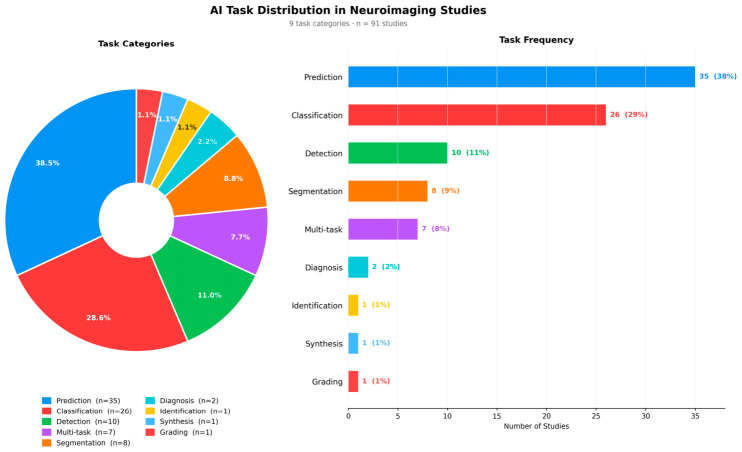
AI task distribution across included neuroimaging studies (*n* = 91, 9 task categories). The donut chart (**left**) illustrates the proportional task distribution. The bar chart (**right**) displays absolute counts and percentages for each task category. Prediction (*n* = 35, 38.5%) and classification (*n* = 26, 28.6%) were the most frequently reported tasks, together accounting for approximately 67% of all included studies.

Prediction was the most frequent task (*n* = 35, 38.5%), followed by classification (*n* = 26, 28.6%). Detection and segmentation were reported in 10 (11.0%) and 8 (8.8%) studies, respectively. Multi-task approaches were used in 7 studies (7.7%). Less common tasks included diagnosis (*n* = 2, 2.2%) and synthesis, identification, and grading (each *n* = 1, 1.1%). Together, prediction and classification accounted for approximately 67.0% of all studies (Figure 6). The detailed characteristics of all included studies are presented in Table 1.

### 3.4. Validation and Dataset Characteristics

**Figure 7:**
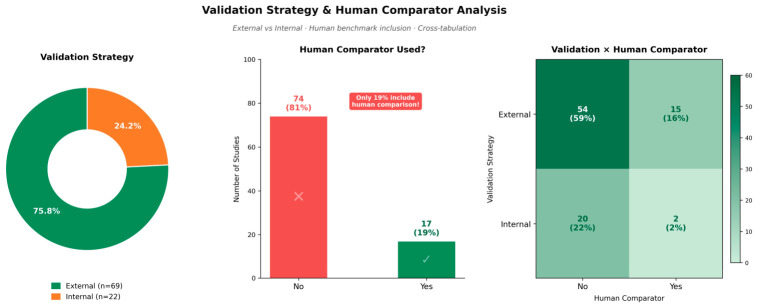
Validation strategy and human comparator analysis (*n* = 91). The donut chart (**left**) illustrates the proportion of studies using external versus internal validation. The bar chart (**center**) shows the frequency of human comparator inclusion. The heatmap (**right**) displays cross-tabulation of validation strategy by human comparator use. Only 19% of studies included a direct human comparator, and this proportion remained low even among externally validated studies.

External validation was performed in 69 studies (75.8%), whereas 22 studies (24.2%) relied solely on internal validation (Figure 7). Cross-tabulation analysis indicated that most studies with external validation did not include comparisons with human experts.

External datasets were used in 66 studies (72.5%), while 25 studies (27.5%) did not use them. This pattern closely aligns with the proportion of studies reporting external validation, with minor differences likely reflecting variations in how external datasets and validation approaches were defined.

Multicenter datasets were the most commonly used, appearing in 60 studies (66.0%). Single-center studies accounted for 22 (24.2%), while 9 studies (9.8%) relied on public datasets. In general, this distribution indicates a significant move towards the use of multi-institutional data sources, and it implies that heterogeneity in the development and validation of AI models should be prioritized.

### 3.5. Methodological Transparency and Bias Indicators

**Figure 8:**
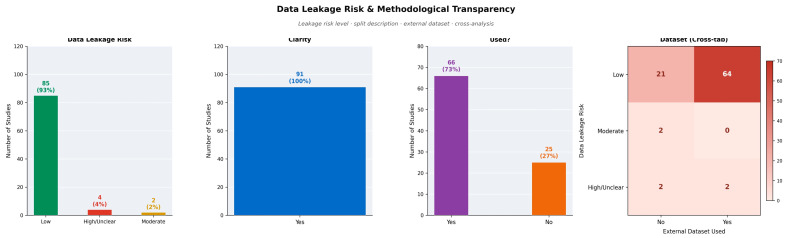
Data leakage risk and methodological transparency analysis (*n* = 91). The bar charts display data leakage risk classification (**left**), split description clarity (**center**-**left**), and use of external datasets (**center**-**right**). The heatmap (**right**) presents cross-tabulation of leakage risk by external dataset use. The majority of studies were classified as low risk (*n* = 85, 93%), and all studies provided clear descriptions of data-splitting procedures.

The majority of the studies (85; 93.4%) were categorized as having a low risk of data leakage based on their reported data-handling and validation procedures. Moderate risk was identified in 2 studies (2.2%), unclear risk in 3 studies (3.3%), and high or potentially unclear risk in 1 study (1.1%). Almost all studies described their data-splitting plans in a transparent manner. Cross-tabulation showed that low leakage risk was most commonly associated with studies using external datasets (Figure 8). As the assessment was based on reported methodology rather than independent verification of practice, subtle forms of leakage not captured in written descriptions cannot be excluded.

Direct comparison with expert human performance was reported in 17 of 91 studies (18.7%); the remaining 74 studies (81.3%) did not include a human benchmark (Figure 7). However, this aggregate rate masks substantial task-dependent heterogeneity (Table 2): 60.0% in detection (6/10), 57.1% in multi-task (4/7), 14.3% in prediction (5/35), 3.8% in classification (1/26), and 0% in segmentation (0/8). The absence of human comparators in segmentation reflects the appropriate use of expert-annotated ground-truth labels, whereas the low rates in classification and prediction tasks that directly inform clinical decision-making represent a more concerning gap than the aggregate figure suggests. Even among externally validated studies, the proportion including a human comparator remained low (Figure 7).

Calibration was reported in 28 of 91 studies (30.8%), with 62 studies (68.1%) not reporting any calibration metric. Upon re-extraction, the most commonly used metric was the calibration curve (*n* = 25, 89.3%), followed by decision curve analysis (*n* = 24, 85.7%), Hosmer–Lemeshow test (*n* = 5, 17.9%), Brier score (*n* = 4, 14.3%), and expected calibration error (*n* = 2, 7.1%). Among studies reporting calibration, 19 (67.9%) assessed calibration on both internal and external validation cohorts, 7 (25.0%) on internal cohorts only, and 2 (7.1%) on external cohorts only. Calibration reporting was ambiguous in one study (1.1%).

The studies showed partial compliance with either CLAIM and/or TRIPOD-AI guidelines, yet none of them were fully compliant, which means that gaps in complete reporting persist (Supplementary Table S2).

The most prevalent reported measure was the area under the receiver operating characteristic curve (AUC), especially when predicting and classifying. The values of AUC were also diverse, with a number of studies showing more than one result in different validation cohorts. Other measures were accuracy, sensitivity, specificity and concordance index (C-index), and Dice similarity coefficient (segmentation). There was a lot of heterogeneity in the definition of outcomes, measures of evaluation, and practices of reporting among studies.

## 4. Discussion

### 4.1. Scope of Included Studies

The present review was deliberately designed with a broad definition of ”neurosurgical relevance” to capture the full methodological landscape of AI imaging studies informing contemporary neurosurgical practice. Beyond intracranial pathology, neurosurgery encompasses spinal surgery, cerebrovascular disease, and increasingly, connectomics-based surgical planning. Accordingly, of the 91 included studies, 86.8% address core intracranial or neurological applications (tumors, stroke, hemorrhage, epilepsy, aneurysms, hydrocephalus), while the remaining 13.2% consist of spinal imaging (8.8%), extracranial cerebrovascular imaging (2.2%), and connectomics or functional imaging applicable to surgical mapping (2.2%). This breadth reflects the multidisciplinary reality of neurosurgical practice and ensures that methodological conclusions regarding validation, calibration, and reporting adherence are drawn from the full spectrum of AI imaging work relevant to the specialty, rather than from a narrower intracranial subset.

The amount of research in AI medical imaging has been increasing significantly over the past few years, and the number of healthcare-related publications per year has risen from 2113 in 2021 to 4587 in 2023, which is more than doubled in two years [102]. Predictive modeling is commonly utilized in various fields of medicine, with oncologic imaging being one of them. In that regard, we performed a dedicated systematic review of AI studies applicable to neurosurgical practice published in 2025 to evaluate the current methodological situation. Instead of subjecting the performance of models to comparison, we intended to assess the methodological rigor and applicability of methods critically and in a real-world setting. We considered the characteristics of datasets (single- vs. multicenter), methods of validation (internal vs. external), transparency of data-splitting processes, and the risk of data leakage. We also evaluated major transparency and quality indicators, such as study design, the use of human comparators, calibration reporting, and compliance with the established frameworks, such as CLAIM and TRIPOD-AI [6,7]. 

The review of 91 studies suggests that there are significant advances; however, limitations that may influence clinical translation remain [3,103,104]. Deep learning methods predominated, and multicenter datasets were commonly employed, indicating increased attention to generalizability. External validation was reported in three-quarters of studies (*n* = 69/91), a marked increase over previous reports, in which external validation was found in only 6–10% of AI medical imaging studies [105,106]. A more recent methodological audit by Spaanderman et al. evaluating AI imaging studies against the CLAIM and FUTURE-AI frameworks through July 2024 similarly identified persistent gaps in external validation reporting; however, direct comparison is limited by differences in clinical scope [107]. This trend reflects growing awareness of the need to test models on independent data.

Regardless of these developments, there are a number of substantial drawbacks. There were few studies (*n* = 17/91) that had direct comparisons with human experts. Though these comparisons might not necessarily be valid, especially with some segmentation or new predictive tasks, the lack of them in research that suggests diagnostic or decision-support uses can restrict the evaluation of added clinical value [103]. Although automated processes may be used, expert monitoring is frequently still required, which highlights the significance of reporting ground-truth quality and inter-rater reliability. Also, almost two-thirds of studies (*n* = 62/91) did not include calibration assessment, which casts doubt on the predictability of the predicted probabilities in clinical decision-making [104].

Even though the majority of the studies were considered to be of low risk of data leakage, discrepancies in the reporting imply that certain methodology problems might still go unnoticed. Moreover, all studies reported partial compliance with CLAIM/TRIPOD-AI guidelines; however, none of them fully complied, and this remains an issue of standardized reporting. Altogether, these results suggest that despite the increase in methodological rigor, there remain major concerns regarding the transparency, validation, and clinical relevance of AI models that prevent their use in a standard clinical environment.

### 4.2. External Validation and Generalizability

The proportion of studies reporting external validation (75.8%) represents a clear improvement over prior literature, in which such validation was typically absent [3,103]. The shift toward external data divides to multicenter clinical cohorts or international competition datasets is a sign that internal validation is an incomplete approach to clinical generalizability [39,59,74].

Nevertheless, external validation does not always ensure strong real-life performance [12]. Numerous investigations used datasets of similar geographically located institutions or processed by similar pipelines [11,15,17,23,31,75,77,80,81,82,83,84,86,87,88,89,90,91,95,96,97,98]. Although this can increase internal consistency, it might not respond well to variation as experienced in normal clinical practice, including variation in scanners, protocols, and patients [39]. The relative lack of experimental studies on true cross-institutional transportability, including between different vendors and workflows, is relatively under-investigated in the literature, even though a few multicenter studies have demonstrated it, including that by Dai M. et al. [40]. Moreover, whereas dataset-level generalizability is gaining more importance, the same cannot be said of human benchmarking or calibration assessment. Consequently, models can be seen to be more generalizable, yet without adequate evaluation to be used in decision support.

A further methodological consideration concerns database coverage in the present review. PubMed was selected as the sole search database to align with the clinical scope of this review, as databases such as EMBASE, Web of Science, and Scopus index a larger proportion of engineering, computer science, and informatics publications that fall outside our predefined clinical focus and were a priori excluded through our eligibility criteria. This choice is consistent with prevailing practice in the field: a recent umbrella review of 158 AI imaging systematic reviews demonstrated that PubMed remains the most frequently used primary search source, appearing in 71.5% of reviews in this area [108]. Nevertheless, single-database searches carry an inherent coverage limitation. Empirical analyses of systematic review database yields have shown that approximately 16% of eligible references may be retrievable from only one database, with EMBASE frequently contributing the largest share of unique references not indexed in MEDLINE/PubMed [109]. Based on these estimates, the expected coverage gap for our PubMed-only search may approximate 5–15% of potentially eligible clinically oriented studies. While this may modestly affect field-level generalizability, the excluded literature would largely consist of engineering-focused work outside the intended scope of this review, making it unlikely that this gap systematically biased our conclusions regarding validation practices, calibration reporting, or guideline adherence.

This restriction is especially critical in the neurosurgical practice, where decisions are made in a multidisciplinary team that involves neurosurgeons, neuroradiologists, and oncologists. Models that are only validated based on performance measures and not on comparison with expert judgment are unable to completely indicate their additional clinical value. In the absence of such benchmarking, claims of clinical applicability are incomplete. In addition to performance measures, the other important but less investigated part of validation is workflow-level evaluation. Even though a recent systematic review identified a significant number of studies that showed an implementation of AI led to a decrease in the time of task completion, workflow impact evaluations are not common [110]. An interesting exception is the clinical trial conducted by Kang et al. [41], which demonstrated better reader performance under AI assistance. On the whole, external validation can be regarded as an indispensable step, but it cannot be considered as being enough. As the field moves towards explainable and clinically integrated AI systems, comprehensive evaluation across multiple dimensions—including transportability, human comparison, calibration, and workflow impact—will be required to establish genuine clinical readiness [42].

### 4.3. Data Leakage and Methodological Transparency

One of the most important but least realized risks to the validity of AI model performance in medical imaging is data leakage. The majority of the studies in this review were rated to be at a low risk of data leakage (*n* = 85/91, 93.4) according to the data handling and partitioning mechanisms reported [43,59] (Supplementary Table S2). Almost all studies presented their training, validation, and test splits explicitly, signifying a higher level of transparency on methodological transparency than the previous literature.

Nevertheless, the completeness and clarity of reporting are critical to assessing the risk of leakage in systematic reviews. Even though the vast majority of studies had separated patient- or center-level datasets, the differences in preprocessing, feature extraction procedures and cross-validation methods can make one less confident about the ability to eliminate less obvious types of leakage. As an example, the less transparent automated pipelines described by Sina et al. were categorized as high/unclear risk because the description lacked adequate detail on internal data partitioning [55]. Similarly, intricate model structures, ambiguous preprocessing procedures, and some feature extraction approaches occasionally blurred dataset boundaries, resulting in moderate or ambiguous risk classifications [42,44,45,92,99]. 

It has been demonstrated in prior studies that even small amounts of preprocessing that occur before splitting datasets can artificially boost model performance in an artificial manner [3,104]. Notably, the large percentage of low-risk studies should be taken with a grain of salt. Data partitioning is not reported clearly enough to rule out the chance of leakage, especially in deep learning pipelines that may include augmentation, normalization, or transfer learning across overlapping data domains. These results emphasize the importance of better reporting of data handling procedures, which are more standardized and detailed, according to the CLAIM and TRIPOD-AI guidelines, to make those reproducible and facilitate the translation of such evidence to a reliable clinical application [7,111]. 

### 4.4. Clinical Benchmarking and Calibration

Despite increasing emphasis on external validation, direct comparison between AI models and human experts remains limited. Only 18.7% of studies (17/91) included a human comparator. However, this aggregate rate masks substantial task-dependent variation (Table 2): 60.0% of detection studies and 57.1% of multi-task studies benchmarked against radiologist performance for clinically actionable tasks, whereas only 14.3% of prediction studies and 3.8% of classification studies incorporated human benchmarking. The 0% rate in segmentation is methodologically appropriate, as segmentation is typically benchmarked against expert-annotated ground-truth labels using metrics such as the Dice coefficient [18,25].

This pattern refocuses the clinical significance of our finding: the scarcity of human benchmarking is not uniform across the field but concentrated in classification and prediction—precisely the task categories where demonstrating clinical added value depends on comparison against expert care. In neurosurgical contexts involving AI-derived probabilistic outputs (e.g., glioma grading, stroke outcome, aneurysm rupture risk), the absence of benchmarking in 96.2% of classification and 85.7% of prediction studies raises serious concerns about clinical translational readiness. Kang et al. provide a notable exception, using a prospective design to directly quantify AI-assisted improvement in clinician performance [41].

Interestingly, in studies that conducted external validation, there was a minimal percentage of studies that proceeded to conduct external validation of their results [38,48,51,52,53,59,61,65]. It implies that, though technical generalizability is becoming more important, meaningful clinical benchmarking has yet to be established. Consequently, assertions of clinical utility can be exaggerated in regard to the actual preparedness of these models for actual application. Whereas measurement of discrimination (e.g., AUC) was consistently reported, calibration assessment was less frequent. Only 30.8% of studies (*n* = 28/91) measured calibration [15,65,66,67,77,83], and almost two-thirds did not measure the alignment of predicted probabilities and observed results [60,92].

Such an imbalance demonstrates a larger pattern in medical AI research, where discrimination is given more importance than probabilistic reliability. The problem is of special interest in the neurosurgical setting, e.g., the glioblastoma prognosis, stroke recovery, or recurrence risk prediction, where probability estimates are themselves directly used by the clinician to make a decision [11,12,14,16,20,21,28,31,34,76,77,78,82,83,86,87,88,89,91,93,95,97]. Such settings have a high chance of poor calibration, resulting in misleading predictions not only in clinical judgment but also in patient counseling.

Notably, despite the high-level of discriminative performance of the models, it is also possible that the outputs of such models are poorly calibrated, which leads to overconfidence or excessive risk stratification. Both discrimination and calibration focus on methodological frameworks like TRIPOD-AI and CONSORT-AI, which underline that a strong model evaluation requires both [7,103]. Enhancement of calibration assessment and reporting is thus most important to achieve safe and effective clinical implementation.

Among the 28 studies reporting calibration (30.8%), calibration curves were the most frequently used metric (89.3%), most often accompanied by decision curve analysis (85.7%); formal calibration statistics such as the Hosmer–Lemeshow test (17.9%) and Brier score (14.3%) were less common, and 67.9% of reporters assessed calibration on both internal and external cohorts. The under-reporting of standard calibration measures, including a calibration curve, Brier scores, or Hosmer-Lemeshow tests, indicates that a lot of modern AI research focuses on comparisons of relative performance, but not on real clinical reliability. In clinical environments, the interpretability and safe application of probability-based predictions can be affected without a regular assessment of calibration. Enhanced reporting of calibration should thus be one of the priorities in order to facilitate sound clinical integration. 

### 4.5. Incomplete Adherence to Standardized Reporting Frameworks

Although there has been an improvement in methodologies, there is still little adherence to established reporting standards. All the studies in this review (*n* = 91) partially adhered to major elements of the CLAIM and /or TRIPOD-AI frameworks and none of them fulfilled them fully (Supplementary Table S2). Although the majority of the studies reported data partitioning and key performance measures unambiguously, other vital aspects, including a description of model development procedures, preprocessing transparency, calibration studies, and implications of clinical implementation, were reported inconsistently.

Such frameworks as CLAIM and TRIPOD-AI have been designed to improve the transparency, reproducibility and interpretability of AI-based prediction research [7,111]. The lack of their full compliance may interfere with the possibility of readers, reviewers, and clinicians to assess the quality of the methodology accurately and determine possible sources of bias. In addition, inadequate reporting can hide such key problems as data leakage, overfitting, or ineffective validation plans.

The lack of complete compliance with all studies reviewed points to the imbalance between accelerated technological development and the slowness of reporting practices development. Enhancing compliance with standardized structures must, therefore, not be considered as a formal requirement, but rather as an essential measure on the way toward better reproducibility, building trust, and making AI models responsible to clinical application.

### 4.6. Translational Maturity and Future Directions

Altogether, these results suggest that AI studies in the field of medical imaging are moving in the direction of more rigorous methods, but the existing evidence does not show that it is ready to be implemented in clinical practice. The increased use of multicenter datasets and external validation is a sign of increased awareness of the necessity of generalizability [40,48,51,59]. Simultaneously, persistent gaps in clinical benchmarking, calibration evaluation, and comprehensive reporting also reveal the major areas that need to be addressed to facilitate the translation between technical development and real-life implementation. Interestingly, this review failed to include research in which prospective clinical implementation or formal workflow impact analysis was conducted, a gap that highlights how the field currently focuses on the development of models rather than implementation science. These findings reflect a contemporaneous snapshot of AI medical imaging publications from 2025, following the 2024 CLAIM and TRIPOD-AI updates, rather than a longitudinal assessment of practice over time.

The fact that human comparator analyses are used sparingly and reporting of calibration metrics is low indicates that most models are tuned to be highly statistical but not necessarily reliable in supporting a decision. The presence of strong discriminatory performance itself is not sufficient to be able to safely integrate into complex neurosurgical and radiological workflows, in which probability estimates directly affect treatment decisions, surgical planning, and prognostic evaluation [72,73]. Even in the absence of strong comparison to expert performance and careful calibration evaluation, the danger of overstating clinical utility is significant.

Also, the lack of complete compliance with standardized reporting frameworks restricts reproducibility and independent validation, which would be necessary to receive regulatory approval and clinical confidence [13,19,20,26,30,32,33,36,37,79,85,90,93]. To be able to make meaningful translational impact, AI models should be evaluated not just in terms of accuracy but also in terms of transparency, reliability, and performance in multidisciplinary clinical settings.

Combined, these findings indicate that the discipline is shifting its focus towards early exploratory development to early phases of translational maturity. The way to attain the desired clinical readiness will be to place an increased emphasis on holistic external validation, strict benchmarking of the end product with human experts, open calibration practice, and the maintenance of accepted reporting standards.

### 4.7. Strengths and Limitations

A number of strengths can be identified with this study. First, it provides a dedicated and current methodological audit of AI imaging studies published in the same year, which allows accurate assessment of the existing validation practices and translational preparation of various medical areas. It is also a method that gives a point of reference in examining how neuroimaging AI has changed as time progresses. Second, a pre-existing, formalized data extraction framework was uniformly used across various areas, such as dataset properties, validation procedure, calibration reporting and compliance with standard guidelines. Third, the risk of data leakage and methodological transparency was systematically defined and evaluated, which made it possible to use cross-study comparisons in a structured manner and not based only on story interpretation.

Nevertheless, a few limitations are to be noted. First, although PubMed was deliberately chosen to align with the clinical focus of this review, the use of a single database may have excluded clinically oriented AI imaging studies published in journals indexed exclusively by EMBASE, Web of Science, or Scopus. This may have introduced a modest selection bias favoring MEDLINE-indexed journals, and our conclusions should therefore be interpreted as characterizing the predominantly clinical AI imaging literature rather than the full multidisciplinary scope of the field.

The review was also restricted to 2025 publications. This year-specific design was chosen to capture the first full publication cycle following the 2024 CLAIM and TRIPOD-AI updates and to enable a contemporaneous audit against current reference standards. Our findings therefore characterize the methodological state of the field at a defined point in time rather than its longitudinal evolution.

Second, adherence to CLAIM and TRIPOD-AI was assessed at the domain level rather than through formal item-level scoring of the 42 CLAIM and 27+ TRIPOD-AI items. Our finding that no study achieved full adherence therefore reflects gaps across major reporting domains rather than precise per-item compliance. Future reviews with a narrower clinical scope could extend this work through formal item-level scoring.

Third, published descriptions were used to classify types of datasets, validation strategies, and risk of data leakage. In situations where reporting was not done fully or clearly, this can cause misclassification, especially in measuring complex or hybrid model architectures. 

Lastly, like any other literature-based review, one cannot eliminate the potential of publication bias. The publications of studies with highly favorable findings or high external validation have a higher chance of publication, and this can result in an overestimation of the methodological quality of the field and its clinical preparedness.

Nevertheless, these restrictions do not invalidate the fact that this review offers a detailed and systematic evaluation of the current validation practices, which have significant gaps that need to be reduced to further advance the clinical translation of AI in medical imaging research.

### 4.8. Future Directions for AI Methodology in Medical Imaging

Further advancements in AI-based medical imaging need to be oriented at going beyond the continued enhancement of model architecture to the enhancement of methodological rigor and clinical integration. External validation must not only progress beyond the replication of datasets but also actual cross-institutional transportability testing, with a variety of scanners, heterogeneous acquisition protocols, and multinational populations of patients. Furthermore, future validation experiments and pragmatic clinical trials will also be necessary to understand whether AI systems have any meaningful contribution to real-world clinical decision-making, but not to improve any statistical measures of performance.

It is also critical to have a regular program of structured benchmarking with human specialists and a regular check of calibration. AI models to be used in decision-support systems should not only exhibit excellent discriminatory results but also robust probability forecasting and evident value addition in multidisciplinary clinical processes. The combination of decision-curve analysis (DCA), clinical impact analysis, and workflow-based analysis will play a significant role in closing the gap between algorithm development and actual clinical utility. 

Moreover, compliance with standardized reporting models like CLAIM and TRIPOD-AI should be considered as the key to reproducibility, regulatory approval, and clinical trust. The current stage of AI development in medical imaging must subsequently focus on transparency, methodological maturity, and evidence-based patient-centered outcomes instead of technical innovation.

## 5. Conclusions

This is a systematic review of AI-based medical imaging studies published in 2025, which shows growing signs of progress in methodological rigor, especially the increased use of multicenter datasets and external validation. Nevertheless, there are continuing weaknesses in clinical benchmarking, calibration evaluation, and overall reporting, which suggest that not all models have been adequately assessed to be used in routine clinical practice. Continued focus on openness, excellent validation, and standard reporting will play a pivotal role in facilitating the safe and successful integration of AI into neurosurgical and radiological practice.

## Figures and Tables

**Table 1:** Summary of included studies: author, country of origin, medical field, specific clinical domain, AI type, AI task (*n* = 91).

Author	Country of Origin	Medical Field	Specific Field	AI Type	AI Task During Research
Akbari H. et al. [59]	Multinational	Oncology	Glioblastoma prognostic subgrouping	ML	Survival prediction and prognostic subgrouping
Albadr R.J. et al. [92]	Multinational	Oncology	Meningioma grading	Hybrid (DL/ML)	Preoperative classification/grading
Belke M. et al. [60]	Germany	Neurology	Epilepsy imaging/hippocampal sclerosis detection	ML	Detection/diagnosis
Cai Z.Y. et al. [75]	China	Neuroscience	White-matter functional connectomics/sex classification	ML	Classification
Chen J. et al. [11]	China	Oncology	Glioblastoma molecular marker prediction (MGMT)	DL	Classification/biomarker prediction
Chen R. et al. [43]	China	Neurosurgery	Intracranial aneurysm outcome prediction	DL	Prediction/risk modeling
Chen Y. et al. [93]	China	Pediatrics	Pediatric brain tumor prognosis	Hybrid (DL/ML)	Prognosis prediction
Chen Y. et al. [12]	USA	Neurology	Intracerebral hemorrhage outcome prediction	DL	Functional outcome prediction
Choi J.H. et al. [94]	S. Korea	Neurosurgery	Intracranial aneurysm rupture prediction	Hybrid (DL/ML)	Classification/rupture risk prediction
Dai M. et al. [40]	Multinational	Radiology	Vertebral compression fracture detection	DL	Detection
Dai Y. et al. [13]	Multinational	Pediatrics	Neonatal hydrocephalus/CSF diversion prediction	DL	Prediction
Demirel E. et al. [61]	Turkey	Oncology	Brain tumor differential diagnosis	ML	Classification
Dong Y. et al. [14]	USA	Neurology/Radiology	Generalizable CTA representation learning for acute stroke tasks	DL	Detection/classification/prediction
Fan Y. et al. [66]	China	Radiology	Pituitary adenoma subtype prediction	ML	Preoperative classification
Fatania K. et al. [62]	UK	Radiology	Glioblastoma radiomics survival modeling	ML	Prognosis modeling
Felefly T. et al. [44]	Multinational	Oncology	Brain metastasis detection on CT	DL	Detection/classification
Feng L. et al. [76]	China	Neurology	Epilepsy surgery outcome prediction	ML	Prediction
Foltyn-Dumitru M. et al. [63]	Germany	Neuroradiology	Glioma imaging phenotyping/survival prediction	ML	Unsupervised clustering/prognosis
Gui Y. et al. [15]	China	Oncology	Meningioma sinus invasion diagnosis	DL	Preoperative classification
Hamon G. et al. [16]	France	Neurology	Synthetic MRI/DWI-FLAIR mismatch assessment	DL	Image synthesis/diagnostic support
Hao M. et al. [58]	China	Oncology	MGMT promoter methylation prediction in glioblastoma	ML	Survival prediction/risk stratification
Harper J.P. et al. [39]	USA	Radiology	Cervical spine fracture detection	DL	Detection
Hossain M.M. et al. [42]	Bangladesh	Neurology	Brain stroke classification on CT	DL	Classification
Hu W. et al. [74]	China	Radiology	Carotid plaque symptom classification	ML	Identification
Huang L. et al. [67]	China	Neurology	Malignant cerebral edema prediction	ML	Prediction
Jeon E.T. et al. [17]	S. Korea	Neurology	White matter hyperintensity/Fazekas grading	DL	Segmentation/grading
Jia X. et al. [38]	China	Neuroradiology	Middle cerebral artery aneurysm rupture risk prediction	DL	Prediction
Kamel P. et al. [18]	USA	Neurology	Ischemic stroke infarct segmentation on MRI	DL	Segmentation
Kang D.W. et al. [41]	S. Korea	Radiology	Intracranial hemorrhage detection	DL	Detection
Kesari A. et al. [19]	India	Oncology	Brain tumor blood-vessel segmentation	DL	Segmentation
Ketabi S. et al. [20]	Canada	Oncology	Pediatric low-grade glioma genetic marker classification	DL	Classification
Kong C. et al. [50]	China	Radiation Oncology	Glioblastoma versus solitary brain metastasis differentiation	DL	Classification
Krag C.H. et al. [21]	Denmark	Neurology	Acute ischemic stroke lesion detection on MRI	DL	Classification
Kulathilake C.D. et al. [22]	Multinational	Neurology	Brain stroke CT classification	DL	Classification
Li D. et al. [95]	China	Oncology	IDH mutation prediction from MRI	Hybrid (DL/ML)	Prediction
Li Z. et al. [96]	China	Radiology	Prediction of stroke recurrence in symptomatic intracranial atherosclerotic stenosis	Hybrid (DL/ML)	Prediction
Liang Q. et al. [68]	China	Oncology	Adult diffuse glioma grading/molecular subtyping	ML	Prediction
Liang X. et al. [23]	China	Oncology	Intracranial solitary fibrous tumor (ISFT) versus angiomatous meningioma differentiation	DL	Classification
Liao L. et al. [46]	France	Neuroradiology	Cerebral aneurysm detection on TOF-MRA	DL	Detection
Lilhore U.K. et al. [24]	India	Oncology	Brain tumor segmentation on multimodal MRI	DL	Segmentation
Lin X. et al. [25]	China	Radiology	Intracranial hemorrhage segmentation on CT	DL	Segmentation
Liu J. et al. [26]	USA	Pediatrics	Prediction of normative pediatric brain development from MRI	DL	Prediction
Liu J. et al. [70]	China	Oncology	MRI-based survival prediction in primary CNS lymphoma	ML	Survival prediction
Liu J. et al. [97]	China	Oncology	Glioblastoma prognostic stratification	Hybrid (DL/ML)	Survival prediction/risk stratification
Lv C. et al. [27]	China	Oncology/Radiology	Brain tumor MRI segmentation	DL	Segmentation
Ma Z. et al. [77]	China	Oncology	MRI radiomics-based classification of malignant brain tumors	ML	Classification
Mahootiha M. et al. [52]	USA	Oncology	Pediatric low-grade glioma recurrence prediction	DL	Prediction/risk modeling
Nada A. et al. [57]	USA	Radiology	Intracranial hemorrhage detection	DL	Detection
Nalentzi K. et al. [45]	Greece	Oncology	Brain tumor MRI classification (glioma versus meningioma)	DL	Classification
Patel B.K. et al. [72]	USA	Oncology	Prediction of extent of resection in giant pituitary neuroendocrine tumors	ML	Prediction
Pelcat A. et al. [28]	France	Neurology	MRI hemorrhage detection in acute stroke	DL	Synthesis
Petterson S. et al. [47]	USA	Neuroradiology	Brain aneurysm detection on CTA	DL	Detection/screening
Rastogi D. et al. [29]	Multinational	Oncology/Radiology	Brain tumor segmentation and survival prediction from MRI	DL	Segmentation/prediction
Roh Y.H. et al. [64]	S. Korea	Neurology	Hemorrhagic transformation prediction in acute ischemic stroke	ML	Prediction/risk modeling
Rühling S. et al. [30]	Germany	Radiology	Osteoporosis screening/bone mineral density analysis	DL	Detection
Ryu W.S. et al. [48]	S. Korea	Neurology	Acute infarct segmentation on MRI	DL	Segmentation
Saadh M.J. et al. [98]	Multinational	Oncology	Meningioma grading	Hybrid (DL/ML)	Classification/grading
Sina E.M. et al. [55]	USA	Otolaryngology	Pituitary macroadenoma vs. parasellar meningioma MRI differentiation	DL	Classification
Song D. et al. [71]	China	Oncology	Atypical meningioma recurrence prediction	ML	Prediction
Sun K. et al. [78]	China	Neurology	Acute ischemic stroke CT radiomics	ML	Radiomics-based detection/classification of MRI-occult ischemic stroke lesions on non-contrast CT
Sun Y. et al. [91]	China	Oncology	Brain metastasis primary tumor origin prediction	ML	Prediction
Sunavsky A. et al. [79]	Canada	Neurology	Chronic low back pain classification using fMRI connectivity	ML	Classification
Topff L. et al. [51]	Netherlands	Oncology	Detection, segmentation, and longitudinal tracking of brain metastases on MRI	DL	Detection, segmentation, and longitudinal tracking
Tu J. et al. [31]	China	Oncology	Glioblastoma infiltration detection in peritumoral edema	DL	Detection/segmentation
Tuxunjiang P. et al. [54]	China	Neurology	Stroke severity prediction using multimodal MRI	DL	Prediction/severity estimation
Wang B. et al. [32]	China	Infectious Disease	MRI differentiation of Brucella and tuberculosis spondylitis	DL	Classification/diagnosis
Wang G. et al. [80]	China	Radiology	Carotid artery stenosis detection on non-contrast CT	ML	Classification/diagnosis
Wang H. et al. [81]	China	Neuroradiology/Stroke	Responsible aneurysm identification in SAH patients with multiple aneurysms	ML	Prediction
Wang H et al. [56]	China	Radiology	ICH black hole sign identification on CT	ML	Prediction
Wang K. et al. [33]	China	Orthopedics	Postoperative outcome prediction after tubular microdiscectomy for lumbar disc herniation	DL	Prediction/outcome classification
Wang T. et al. [34]	China	Radiology/Stroke	Post-thrombectomy intracranial hemorrhage CT differentiation	DL	Image generation/diagnostic classification
Wang Y. et al. [35]	China	Oncology	Brain metastasis segmentation	DL	Segmentation
Xia X. et al. [82]	China	Oncology	Glioblastoma versus solitary brain metastasis differentiation	ML	Classification/diagnosis
Xia X. et al. [69]	China	Neurology	Functional outcome prediction after ICH	ML	Prediction/prognosis
Xing L. et al. [36]	China	Orthopedics/Spine	Modic changes detection and grading on lumbar spine MRI	DL	Detection/grading
Xu W. et al. [83]	China	Oncology	Grade 4 glioma molecular subtyping with MRI radiomics	ML	Preoperative molecular subtype classification and prognostic stratification
Xu X. et al. [84]	China	Neurology	Prediction of cerebrovascular disease related cognitive impairment	ML	Prediction/risk stratification
Yang H. et al. [49]	China	Neurology	Prognostic prediction in acute ischemic stroke after thrombolysis	DL	Prediction/prognosis
Yang Q. et al. [65]	China	Oncology	Pituitary neuroendocrine tumor consistency prediction using mpMRI radiomics	ML	Classification/prediction
Ye B. et al. [85]	China	Orthopedics/Spine	Prediction of vertebral artery injury during C2 pedicle screw placement	ML	Risk prediction/classification
Yin L. et al. [99]	China	Oncology/Radiology	Preoperative glioma grading using MRI	Hybrid (DL/ML)	Classification/diagnosis
Yin S. et al. [100]	China	Oncology	Preoperative glioma grading	Hybrid (DL/ML)	Classification/grading
Yonar A. et al. [101]	Turkey	Oncology	Brain tumor type classification using MRI	Hybrid (DL/ML)	Classification/diagnosis
Zahoora U. et al. [37]	Pakistan	Oncology	Brain tumor segmentation on MRI	DL	Segmentation
Zeng L. et al. [53]	China	Neuroradiology	Intracranial aneurysm stability prediction on CTA	DL	Classification/risk prediction
Zeng Q. et al. [86]	China	Oncology	Glioblastoma versus solitary brain metastasis differentiation	ML	Classification/diagnosis
Zhai D. et al. [87]	China	Neuroradiology/Stroke	Hemorrhagic transformation versus contrast extravasation differentiation after mechanical thrombectomy	ML	Classification/diagnosis
Zhao K. et al. [89]	China	Oncology	Differential analysis between PCNSL versus low grade glioma	ML	Classification
Zhao K. et al. [88]	China	Oncology	Pituitary adenoma Ki-67 prediction	ML	Prediction
Zheng B. et al. [73]	China	Neurosurgery	Cervical spondylotic myelopathy prognosis prediction	ML	Prediction
Zhuang X. et al. [90]	China	Orthopedics	Spine fracture imaging analysis	ML	Classification

Abbreviations: AI, artificial intelligence; ML, machine learning; DL, deep learning; Hybrid (DL/ML), hybrid deep learning and machine learning. Full data extraction, including study aims, main conclusions, detailed methodological variables, and additional abbreviations, is provided in Supplementary Table S1.

**Table 2:** Human comparator inclusion stratified by AI task category (*n* = 91).

AI Task Category	Total (*n*)	With Human Comparator (*n*)	%	Clinical Interpretation
Classification	26	1	3.8%	Critical gap—benchmarking against clinicians essential for diagnostic AI
Prediction	35	5	14.3%	Substantial gap—benchmarking against clinical scores/experts needed
Detection	10	6	60.0%	Adequate—most detection systems benchmarked against radiologists
Segmentation	8	0	0.0%	Methodologically appropriate—Dice coefficient vs. expert ground-truth
Multi-task	7	4	57.1%	Adequate—mostly driven by detection sub-components
Other *	5	1	20.0%	-
Total	91	17	18.7%	

* Other includes Diagnosis (*n* = 2), Identification (*n* = 1), Grading (*n* = 1), Synthesis (*n* = 1).

## Data Availability

The data presented in this study are available on request from the corresponding author.

## Supplementary Materials

The following supporting information can be downloaded at: https://www.mdpi.com/article/10.3390/jcm15093441/s1, Supplementary Data S1: Detailed search strategy; Supplementary Table S1: Summary of included studies; Supplementary Table S2: Full data extraction of included studies; Supplementary Table S3: Risk of bias assessment of included studies using PROBAST+AI; PRISMA 2020 checklist.

## Abbreviations

The following abbreviations are used in this manuscript:

AI	Artificial intelligence
AUC	Area under the receiver operating characteristic curve
C-index	Concordance index
CLAIM	Checklist for Artificial Intelligence in Medical Imaging
CONSORT-AI	Consolidated Standards of Reporting Trials for Artificial Intelligence
CT	Computed tomography
CTA	Computed tomography angiography
DCA	Decision-curve analysis
DL	Deep learning
ML	Machine learning
MRA	Magnetic resonance angiography
MRI	Magnetic resonance imaging
PRISMA	Preferred Reporting Items for Systematic Reviews and Meta-Analyses
PROBAST+AI	Prediction model Risk of Bias Assessment Tool for Artificial Intelligence
PROSPERO	International Prospective Register of Systematic Reviews
TRIPOD-AI	Transparent Reporting of a multivariable prediction model for Individual Prognosis or Diagnosis using Artificial Intelligence
