# Supplementary material for: Are AI Neuroimaging Models Ready for Clinical Use? A Systematic Methodological Review
Source: J Clin Med. 2026 Apr 30;15(9):3441. doi: 10.3390/jcm15093441 (PMC13164521; doi:10.3390/jcm15093441)
Supplement: Supplementary file 1 [file jcm-15-03441-s001.zip › Supplemental DATA S1.pdf]

**Article title**: **Are AI Neuroimaging Models Ready for Clinical Use? A Systematic Methodological Review**

**Journal:** Journal of Clinical Medicine
**Authors:** Umid Sulaimanov, Nafiye Sanlier, Ariorad Moniri, Behman Demir, Yerkebulan Serikkanov, Ahmed Rasim Bayramoglu, Maryam Sabah Al-Jebur, Irem Uslu, Oyku Ozturk, Mariagrazia Nizzola, Erkin Ötleş, Simon Ammanuel, Abdullah Keles, Ufuk Erginoglu, Mustafa K. Baskaya*

**Corresponding author:** Mustafa K. Baskaya

**Supplementary Data:** Detailed search strategy used for all databases

| | |
|---|---|
| #1 | ( "Artificial Intelligence"[MeSH] OR "Machine Learning"[MeSH] OR "Neural Networks, Computer"[MeSH] OR artificial intelligence[Title/Abstract] OR machine learning[Title/Abstract] OR deep learning[Title/Abstract] OR neural network*[Title/Abstract] OR radiomics[Title/Abstract] |
| #2 | ( "Diagnostic Imaging"[MeSH] OR medical imaging[Title/Abstract] OR radiology[Title/Abstract] OR MRI[Title/Abstract] OR CT[Title/Abstract] ) |
| #3 | ( bias[Title/Abstract] OR "external validation"[Title/Abstract] OR generalizability[Title/Abstract] OR reproducibility[Title/Abstract] OR "data leakage"[Title/Abstract] ) AND "Humans"[MeSH] |
| #4 | ("2025/01/01"[Date - Publication] : "2025/12/31"[Date - Publication]) |
| #5 | #1 AND #2 AND #3 AND #4 |

**Supplementary Data 1**.Search strategy
