# Supplementary material for: Are AI Neuroimaging Models Ready for Clinical Use? A Systematic Methodological Review
Source: J Clin Med. 2026 Apr 30;15(9):3441. doi: 10.3390/jcm15093441 (PMC13164521; doi:10.3390/jcm15093441)
Supplement: Supplementary file 1 [file jcm-15-03441-s001.zip › Supplementary Table S1.pdf]

Table 1. Summary of included studies: author, country of origin, medical field, specific clinical domain, AI type, AI task, study aim, and main conclusion (n = 91).

**Author**	Country of Origin	Medical Field	Specific Field	AI Type	AI Task During Research	Aim of Research	Conclusion of Research
Akbari H. et al.[59]	Multi-national	Oncology	Glioblastoma prognostic subgrouping	ML	Survival prediction and prognostic subgrouping	To develop a reproducible machine-learning framework integrating routine clinical data and MRI features to stratify glioblastoma patients into prognostic subgroups across a large multicenter cohort	The machine-learning model successfully stratified glioblastoma patients into distinct prognostic subgroups across a large, multi-institutional cohort, demostrating its potential for reproducible prognostic assessment and clinical decision support
Albadr R.J. et al.[92]	Multi-national	Oncology	Meningioma grading	Hybrid (DL/ML)	Preoperative classification/grading	To develop a clinically applicable MRI framework integrating radiomic and deep learning features for preoperative meningioma grading	Integrating radiomics, deep features, and attention mechanisms significantly improved grading accuracy and reproducibility, demonstrating strong potential for reliable preoperative meningioma assessment
Belke M. et al.[60]	Germany	Neurology	Epilepsy imaging/hippocampal sclerosis detection	ML	Detection/diagnosis	To develop and validate an AI-enhanced tool for automatic detection of hippocampal sclerosis from routine MRI acquired across different scanners and protocols	The automated system accurately detected hippocampal sclerosis across heterogeneous routine MRI data, demonstrating robustness and potential utility in clinical epilepsy evaluation
Cai Z.Y. et al.[75]	China	Neuroscience	White-matter functional connectomics/sex classification	ML	Classification	To investigate sexual dimorphism in white-matter functional connectomes and evaluate whether these features can predict biological sex	White-matter and gray-matter/white-matter functional connectivity exhibited significant sex-related differences, and SVM-based classification demonstrated moderate predictive ability for sex identification

Chen J. et al.[11]	China	Oncology	Glioblastoma molecular marker prediction (MGMT)	DL	Classification/biomarker prediction	To evaluate whether multiparametric MRI combined with off-the-shelf deep learning features can noninvasively predict MGMT promoter methylation status in glioblastoma	The proposed deep-feature framework demostrated the feasibility of noninvasive MGMT promoter methylation prediction from mpMRI and showed reliable performance across datasets, supporting further development toward clinical application
Chen R. et al.[43]	China	Neurosurgery	Intracranial aneurysm outcome prediction	DL	Prediction/risk modeling	To develop a DWI-based deep learning radiomics nomogram to predicts impaired health-related quality of life after stent placement in aneurysm patients with iatrogenic infarction	The integrated clinical, radiomic, and deep learning nomogram outperformed single-modality models and demonstraed robust predictive performance for HRQOL outcomes across cohorts
Chen Y. et al.[93]	China	Pediatrics	Pediatric brain tumor prognosis	Hybrid (DL/ML)	Prognosis prediction	To develop a multimodal machine-learning model integrating MRI radiomics, deep learning features, and clinical variables to predict 3-year postoperative disease-free survival in pediatric malignant brain tumors	The multimodal fusion model integrating radiomics, deep learning features, and clinical variables achieved superior prognostic performance compared with single-modality models, demonstrating strong potential for postoperative risk stratification in pediatric brain tumor
Chen Y. et al.[12]	USA	Neurology	Intracerebral hemorrhage outcome prediction	DL	Functional outcome prediction	To develop a deep survival model that predicts long-term functional outcomes after intracerebral hemorrhage directly from admission non-contrast CT scans	The deep learning survival model achieved higher predictive performance than conventional prognostic scores (ICH and FUNC) for long-term functional outcomes after intracerebral hemorrhage, demonstrating potential clinical utility for post-ICH prognostication

Choi J.H. et al.[94]	S. Korea	Neurosurgery	Intracranial aneurysm rupture prediction	Hybrid (DL/ML)	Classification/rupture risk prediction	To develop a fully automated AI framework integrating deep learning segmentation and machine learning models to predict intracranial aneurysm rupture status from CTA-derived vascular features	The automated framework demostrated strong predictive performance and may assist clinicians in intracranial aneurysm rupture risk assessment
Dai M. et al.[40]	Multi-national	Radiology	Vertebral compression fracture detection	DL	Detection	To validate a deep-learning tool for incidental vertebral compression fracture detection on routine CT across multiple sites and vendors.	The algorithm demostrated high accuracy, sensitivity, and specificity across sites, supporting its use for opportunistic fracture detection in routine CT
Dai Y. et al.[13]	Multi-national	Pediatrics	Neonatal hydrocephalus/CSF diversion prediction	DL	Prediction	To develop a multimodal AI model integrating MRI and clinical variables to predict the need for cerebrospinal fluid diversion in neonates with hydrocephalus.	The multimodal model outperformed image-only and clinical-only approaches and demonstrated generalization in external validation, suggesting potential for treatment stratification in neonatal hydrocephalus
Demirel E. et al.[61]	Turkey	Oncology	Brain tumor differential diagnosis	ML	Classification	To distinguish high-grade (grade 4) glioma from brain metastasis using machine learning models based on radiomic features extracted from peritumoral edema on T2-FLAIR subtraction images	Radiomics-based machine learning demonstrated excellent discrimination between high-grade glioma and brain metastasis, suggesting potential utility for preoperative tumor classification
Dong Y. et al.[14]	USA	Neurology/Radiology	Generalizable CTA representation learning for acute stroke tasks	DL	Detection/classification/prediction	To develop a generalizable self-supervised learning framework for brain CTA that can support multiple acute stroke	Self-supervised pretraining improved performance across several stroke-related CTA tasks and enhanced model generalizability compared with standard supervised learning

						tasks using largely unlabeled data.	
Fan Y. et al.[66]	China	Radiology	Pituitary adenoma subtype prediction	ML	Preoperative classification	To develop a multiparametric MRI-based clini-radiomic model to preoperatively differentiate sparsely and densely granulated somatotroph adenomas	The clini-radiomic model demonstrated excellent discrimination with external multicentric validation, supporting noninvasive preoperative subtype prediction of somatotroph adenomas
Fatania K. et al.[62]	UK	Radiology	Glioblastoma radiomics survival modeling	ML	Prognosis modeling	To assess how MRI intensity standardization techniques and ComBat batch size influence the performance and stability of radiomics-based survival models in multicenter glioblastoma MRI dataset	Intensity standardization and ComBat harmonization improved discrimination and model fit in some settings, but larger batch sizes reduced stability and calibration, highlighting reproducibility trade-offs in multicenter radiomics studies
Felefly T. et al.[44]	Multi-national	Oncology	Brain metastasis detection on CT	DL	Detection/classification	To develop a 3D convolutional neural network using non-enhanced brain CT to distinguish patients with and without brain metastases	The 3D-CNN achieved very high validation accuracy in distinguishing patients with and without brain metastases, warranting external validation
Feng L. et al.[76]	China	Neurology	Epilepsy surgery outcome prediction	ML	Prediction	To evaluate individualized structural covariance network abnormalities on preoperative MRI and develop a machine-learning model to predict postoperative seizure outcomes in mesial temporal lobe epilepsy	Contralateral network abnormalities were strongly associated with non-seizure-free outcomes, and the model generalized well across external datasets

Foltyn-Dumitru M. et al.[63]	Germany	Neuroradiology	Glioma imaging phenotyping/survival prediction	ML	Unsupervised clustering/prognosis	To identify MRI shape-radiomic phenotypes in IDH-wildtype glioma using unsupervised clustering and evaluate their association with overal survival	Unsupervised shape-radiomic clustering identified phenotypes with significantly different survival and improved prognostic performance beyond tumor volume alone.
Gui Y. et al.[15]	China	Oncology	Meningioma sinus invasion diagnosis	DL	Preoperative classification	To build a fusion model combining MRI radiomics and deep learning features for preoperative diagnosis of meningioma sinus invasion.	The combined radiomics-deep learning model outperformed single-feature models and showed value for preoperative assessment of sinus invasion.
Hamon G. et al.[16]	France	Neurology	Synthetic MRI/DWI-FLAIR mismatch assessment	DL	Image synthesis/diagnostic support	To evaluate whether a deep learning generated synthetic FLAIR can substitute real FLAIR for DWI-FLAIR mismatch assessment and early acute ischemic stroke detection in a multicenter cohort	Synthetic FLAIR demonstrated high concordance with real FLAIR and comparable sensitivity and specificity for identifying early ischemic stroke
Hao M. et al.[58]	China	Oncology	MGMT promoter methylation prediction in glioblastoma	ML	Survival prediction/risk stratification	To develop and validate an MRI radiomics model combined with clinical and molecular biomarkers for predicting overal survival in glioma patients	Radiomics-based models showed strong performance for predicting overal survival and effectively stratified glioma patients into high- and low-risk groups
Harper J.P. et al.[39]	USA	Radiology	Cervical spine fracture detection	DL	Detection	To externally validate a top-performing deep learning algorithm from the RSNA 2022 Cervical Spine Fracture Detection Challenge using an independent clinical CT dataset	The deep learning model achieved good external performance, demonstrating potential as an emergency triage tool for cervical spine fracture detection, although specificity was lower in the external cohort

Hossain M.M. et al.[42]	Bangladesh	Neurology	Brain stroke classification on CT	DL	Classification	To develop a deep learning model combining Vision Transformer and LSTM for classification of brain stroke from CT images	The proposed ViT-LSTM deep learning model achieved high classification accuracy and demonstrated improved performance for CT-based brain stroke classification
Hu W. et al.[74]	China	Radiology	Carotid plaque symptom classification	ML	Identification	This study aims to create a radiomics nomogram using dual-energy computed tomography (DECT) virtual monoenergetic images (VMI) to accurately identify symptomatic carotid plaques.	Despite the successful use of DECT radiomics in oncology, its potential application in arterial plaque assessment remains largely unexplored. The presented study addresses this gap by introducing a highly accurate DECT-based radiomics nomogram to identify symptomatic carotid plaques
Huang L. et al.[67]	China	Neurology	Malignant cerebral edema prediction	ML	Prediction	To develop and validate a multimodal model integrating clinical, radiological, and radiomic features based on the ASPECTS framework to predict malignant cerebral edema after acute ischemic stroke	The multimodal fused model showed superior predictive performance and may support early risk estimation on malignant cerebral edema
Jeon E.T. et al.[17]	S. Korea	Neurology	White matter hyperintensity/Fazekas grading	DL	Segmentation/grading	To develop a deep learning pipeline for automated WMH segmentation and Fazekas scale grading using spatial-probabilistic model from FLAIR MRI	The proposed deep learning pipeline demostrated robust performance for automated segmentation of white matter hyperintensities and accurate Fazekas scale grading using FLAIR MRI alone, showing strong agreement with expert ratings and good generalizability across validation datasets

Jia X. et al.[38]	China	Neuroradiology	Middle cerebral artery aneurysm rupture risk prediction	DL	Prediction	To develop a multimodal deep learning model integrating CTA images, radiomics features, and clinical-morphological data to predict rupture risk in middle cerebral artery aneurysms	Multimodal integration significantly improved rupture prediction performance, and the proposed MCANet model demonstrated high accuracy with successfull external validation, indicating potential clinical applicability for individualized rupture risk assessment
Kamel P. et al.[18]	USA	Neurology	Ischemic stroke infarct segmentation on MRI	DL	Segmentation	To evaluate deep learning models for ischemic stroke infarct segmentation on MRI and compare the performance of self-configuring nnU-Net with standart U-Net architectures across different MRI sequences	Self-configuring nnU-Net models achieved high performance for infarct segmentation and significantly outperformed standart U-Net architectures, demonstrating robust performance on external clinical datasets
Kang D.W. et al.[41]	S. Korea	Radiology	Intracranial hemorrhage detection	DL	Detection	To validate a deep learning algorithm (JLK-ICH) for intracranial hemorrhage detection on CT and evaluate its clinical utility in improving reader diagnostic performance	The deep learning algorithm demonstrated high accuracy in detecting ICH and improved the diagnostic performance of residents when interpreting CT scans
Kesari A. et al.[19]	India	Oncology	Brain tumor blood-vessel segmentation	DL	Segmentation	To develop a deep learning framework for automated segmentation of low-intensity blood vessels within brain tumor images to improve quantitative vascular analysis	The proposed deep learning framework achieved accurate segmentation of low-intensity tumor blood vessels and outperformed existing approaches, demostrating potential for improved quantitative analysis of tumor vasculature

Ketabi S. et al.[20]	Canada	Oncology	Pediatric low-grade glioma genetic marker classification	DL	Classification	To develop multimodal contrastive learning framework integrating MRI and radiology reports to improve explainability and performance of pediatric low-grade. Glioma genetic marker classification	The proposed MRI-report contrastive learning framework improved the performance and explainability of pediatric low-grade glioma genetic marker classification compared with baseline models
Kong C. et al.[50]	China	Radiation Oncology	Glioblastoma versus solitary brain metastasis differentiation	DL	Classification	To develop and compare deep learning models based on multi-sequence MRI for preoperative differentiation of glioblastoma and solitary brain metastasis	Deep learning models based on multi-sequence MRI can accurately differentiate glioblastoma from solitary brain metastasis, with 3D ResNet-18 demonstrating the best performance
Krag C.H. et al.[21]	Denmark	Neurology	Acute ischemic stroke lesion detection on MRI	DL	Classification	To evaluate spectrum bias in deep learning based MRI analysis for acute ischemic stroke by examining the impact of excluding uncertain ischemic lesions	Excluding uncertain ischemic lesions leads to substantial overestimation of diagnostic performance, highlighting the importance of accounting for spectrum bias in validation of AI-based stroke imaging models
Kulathilake C.D. et al.[22]	Multi-national	Neurology	Brain stroke CT classification	DL	Classification	To develop and validate deep learning models using CT imaging for classification of stroke types and ischemic stroke stages	Deep learning models demonstrated high accuracy, in classifying stroke types and ischemic stroke stages from CT images, suggesting potential utility for clinical decision support
Li D. et al.[95]	China	Oncology	IDH mutation prediction from MRI	Hybrid (DL/ML)	Prediction	To develop and validate a deep learning radiomics nomogram for noninvasive prediction of IDH mutation status in glioma using MRI	The deep learning radiomics nomogram demostrated good performance for noninvasive prediction of IDH mutation status in glioma and may assist in personalized treatment planning

Li Z. et al.[96]	China	Radiology	Prediction of stroke recurrence in symptomatic intracranial atherosclerotic stenosis	Hybrid (DL/ML)	Prediction	To develop and evaluate a 3D deep learning model based on HR-VWI for predicting recurrence risk in patients with symptomatic intracranial atherosclerotic stenosis	The 3D deep learning model based on HR-VWI demonstrated superior performance for predicting symptomatic intracranial atherosclerotic stenosis recurrence risk compared with 2D deep learning and radiomics models
Liang Q. et al.[68]	China	Oncology	Adult diffuse glioma grading/molecular subtyping	ML	Prediction	To develop and validate radiopathomics models integrating MRI radiomics and pathomics features to predict molecular subtypes and WHO grades in adult diffuse gliomas	Radiopathomics models integrating radiomics and pathomics features demonstrated superior performance for predicting molecular subtypes and WHO grades of adult diffuse gliomas compared with single-modality approaches
Liang X. et al.[23]	China	Oncology	Intracranial solitary fibrous tumor (ISFT) versus angiomatous meningioma differentiation	DL	Classification	To develop and validate an MRI-based deep learning radiomic nomogram to distinguish intracranial solitary fibrous tumors from angiomatous meningiomas and predict overal survival in ISFT patients	The MRI-based deep learning radiomic nomogram outperformed the clinical model in distinguishing ISFTs from angiomatous meningiomas and demostrated prognostic value for predicting overal survival
Liao L. et al.[46]	France	Neuroradiology	Cerebral aneurysm detection on TOF-MRA	DL	Detection	To evaluate the performance of an AI system for detecting cerebral aneurysms on 3D TOF-MRA and assess its impact on radiologist detection accuracy and inter-reader variablility	AI assistance improved the detection of cerebral aneurysms on TOF-MRA and reduced inter-reader variability among radiologists
Lilhore U.K. et al.[24]	India	Oncology	Brain tumor segmentation on multimodal MRI	DL	Segmentation	To develop a deep learning framework with attention mechanisms and domain adaptation for accurate segmentation of brain	The proposed deep learning framework achieved improved brain tumor segmentation performance on multimodal MRI and demostrated robust generalization across datasets

						tumors from multimodal MRI scans	
Lin X. et al.[25]	China	Radiology	Intracranial hemorrhage segmentation on CT	DL	Segmentation	To develop and evaluate an attention-based residual U-Net model for accurate multi-label segmentation of intracranial hemorrhage type on CT images	The attention-based residual U-Net achieved higher multi-labeled segmentation accuracy than conventional U-Net models, demonstrating potential for automated intracranial hemorrhage analysis in clinical settings
Liu J. et al.[26]	USA	Pediatrics	Prediction of normative pediatric brain development from MRI	DL	Prediction	To develop a deep learning predicting normative pediatric brain growth tragectories from neuroimaging data	The proposed deep learning model accurately predicts normative pediatric brain growth trajectories and may facilitate early identification of developmental abnormalities
Liu J. et al.[70]	China	Oncology	MRI-based survival prediction in primary CNS lymphoma	ML	Survival prediction	To develop and validate an MRI-based radiomics model combined with clinical factors to predict progression-free survival (PFS) and overall survival (OS) of patients with primary CNS lymphoma	The radiomics model demonstrated greater predictive value than clinical factors alone, and the combined radiomics-clinical model achieved the best performance for predicting survival outcomes in primary CNS lymphoma patients
Liu J. et al.[97]	China	Oncology	Glioblastoma prognostic stratification	Hybrid (DL/ML)	Survival prediction/risk stratification	To construct and validate an MRI-based radiomics machine learning model for predicting overall survival in IDH-wildtype glioblastoma after maximal safe surgical resection using MRI	The deep learning segmentation-based radiomics model demonstrated robust performance in predicting overal survival in IDH-wildtype glioblastoma and may support personalized prognostic assessment

Lv C. et al.[27]	China	Oncology/Radiology	Brain tumor MRI segmentation	DL	Segmentation	To enhance the robustness of the Segment Anything Model (SAM) for medical image segmentation by proposing the SAM-RCCF framework and evaluating its performance on brain tumor MRI segmentation tasks	The SAM-RCCF framework significantly improved segmentation performance compared with the original SAM across glioma, metastatic tumor, and meningioma MRI datasets
Ma Z. et al.[77]	China	Oncology	MRI radiomics-based classification of malignant brain tumors	ML	Classification	To construct a three-class multiparametric MRI radiomics model incorporating diffusion-weighted imaging to classify supratentorial malignant brain tumors	DWI-based radiomic features provided incremental diagnostic value and improved the performance of models distinguishing high grade glioma, brain metastases, and primary CNS lymphoma
Mahootiha M. et al[52]	USA	Oncology	Pediatric low-grade glioma recurrence prediction	DL	Prediction/risk modeling	To develop a multimodal deep learning model integrating imaging and clinical data to predict recurrence risk in pediatric low-grade glioma	The multimodal deep learning model integrating imaging and clinical features improved prediction of recurrence risk in pediatric low-grade glioma compared with conventional approaches
Nada A. et al.[57]	USA	Radiology	Intracranial hemorrhage detection	DL	Detection	To externally validate and evaluate the performance of an FDA-approved deep learning model for detecting intracranial hemorrhage (ICH) on a real-world clinical CT dataset	The deep learning model demonstrated strong performance for intracranial hemorrhage detection on a heterogeneous real-word dataset and showed potential as a supportive tool in clinical radiology workflow
Nalentzi K. et al.[45]	Greece	Oncology	Brain tumor MRI classification (glioma versus meningioma)	DL	Classification	To compare the performance of transformer-based deep learning models (ViT and BEiT) for MRI-based classification of gliomas and meningiomas using a	The proposed feature-driven framework demonstrated improved classification accuracy and robustness for distinguishing gliomas and meningiomas using transformer-based deep learning models

						feature-driven methodology	
Patel B.K. et al.[72]	USA	Oncology	Prediction of extent of resection in giant pituitary neuroendocrine tumors	ML	Prediction	To develop predictive model using preoperative imaging parameters to estimate the extent of resection in giant pituitary neuroendocrine tumors	Three-dimentional preoperative imaging parameters provide valuable predictive information for estimating the extent of resection in giant pituitary neuroendocrine tumors and may assist surgical planning
Pelcat A. et al.[28]	France	Neurology	MRI hemorrhage detection in acute stroke	DL	Synthesis	To validate a deep learning algorithm that generates synthetic T2-weighted images from diffusion-weighted MRI and compare its diagnostic performance with true T2-weighted images for hemorrhage detection in acute stroke	Synthetic T2-weighted images generated by deep learning showed similar diagnostic performance to true T2-weighted images for hemorrhage detection and may help shorten MRI protocols in acute stroke imaging
Petterson S. et al.[47]	USA	Neuroradiology	Brain aneurysm detection on CTA	DL	Detection/screening	To develop and externally validate a deep learning model for automated detection of intracranial aneurysm on CT angiography with improved generalizability	Deep learning-based aneurysm detection demonstrated high sensitivity and specificity with successful international external validation, highlighting improved model generalizability and enabling broader testing through a web-based platform.
Rastogi D. et al.[29]	Multi-national	Oncology/Radiology	Brain tumor segmentation and survival prediction from MRI	DL	Segmentation/prediction	To develop deep learning framework for brain tumor feature extraction and segmentation from MRI image and to predict patient survival days using predictive deep learning models	The proposed deep learning framework effectively extracts tumor features, segments brain tumors on MRI, and predicts patient survival days, demonstrating promising performance for glioma prognosis analysis

Roh Y.H. et al.[64]	S. Korea	Neurology	Hemorrhagic transformation prediction in acute ischemic stroke	ML	Prediction/risk modeling	To develop and validate a clinico-radiomics model integrating multiparametric MRI radiomic features and clinical scoring systems to predict hemorrhagic transformation in acute ischemic stroke	Clinico-radiomics models integrating MRI radiomic features and clinical variables improved prediction of hemorrhagic transformation compared with clinical models alone
Rühling S. et al.[30]	Germany	Radiology	Osteoporosis screening/bone mineral density analysis	DL	Detection	To evaluate the accuracy of opportunistic measurements of volumetric bone mineral density (vBMD) in intraoperative multi-detector CT (MDCT) scans, using preoperative MDCT as the reference.	Opportunistic osteoporosis screening with the presented approach is feasible and demonstrates high accuracy in reference to preoperative MDCT scans.
Ryu W.S. et al.[48]	S. Korea	Neurology	Acute infarct segmentation on MRI	DL	Segmentation	To develop and validate a deep learning model for automatic segmentation of cerebral infarcts on diffusion-weighted MRI	The deep learning model achieved reliable automatic segmentation of cerebral infarcts on diffusion MRI and demonstrated similar performance in external validation, although performance was lower for very small lesions
Saadh M.J. et al.[98]	Multi-national	Oncology	Meningioma grading	Hybrid (DL/ML)	Classification/grading	To develop a reliable framework for preoperative meningioma grading by intergrating radiomic features with deep deep learning features extracted from a 3D autoencoder	The integrated framework combining radiomic and deep learning features provides a robust, noninvasive, and reproducible approach for preoperative meningioma grading

Sina E.M. et al.[55]	USA	Otolaryngology	Pituitary macroadenoma vs parasellar meningioma MRI differentiation	DL	Classification	To evaluate automated machine learning methods to differentiate pituitary macroadenomas from parasellar meningiomas on preoperative MRI	The AutoML models showed high diagnostic accuracy and may assist radiologists in preoperative tumor differentiation
Song D. et al.[71]	China	Oncology	Atypical meningioma recurrence prediction	ML	Prediction	To investigate factors associated with recurrence in atypical meningioma and develop a predictive model integrating radiomic and clinical features	Radiomics has additional value for predicting AM tumor recurrence and has favorable predictive performance when combined with clinical features.
Sun K. et al.[78]	China	Neurology	Acute ischemic stroke CT radiomics	ML	Radiomics-based detection/classification of MRI-occult ischemic stroke lesions on non-contrast CT	To develop and validate a non-contrast CT radiomics plus machine learning approach for detecting acute ischemic stroke lesions that are not visible to radiologists but are confirmed on DWI MRI.	Radiomics combined with machine learning provided reliable discrimination of microscopic acute ischemic stroke on non-contrast CT and could assist radiologists in early clinical decision-making.
Sun Y. et al.[91]	China	Oncology	Brain metastasis primary tumor origin prediction	ML	Prediction	To investigate whether habitat-based radiomics can identify the metastatic tumor type of BM and whether an imaging-based model that integrates the volume of peritumoral edema (VPE) can enhance predictive performance.	The developed habitat-based radiomics models can effectively identify the metastatic tumor type of BM and may be considered as a potential preoperative basis for timely treatment planning.
Sunavsky A. et al.[79]	Canada	Neurology	Chronic low back pain classification using fMRI connectivity	ML	Classification	To investigate whether nucleus accumbens-prefrontal cortex functional connectivity can serve as a biomarker for classifying chronic low back pain using	The study demonstrates that abnormal nucleus accumbens-prefrontal connectivity is associated with chronic low back pain and can be used by machine learning classifiers to

						machine learning approaches	distinguish chronic low back pain from healthy individuals
Topff L. et al.[51]	Netherlands	Oncology	Detection, segmentation, and longitudinal tracking of brain metastases on MRI	DL	Detection, segmentation, and longitudinal tracking	To develop a generalizable deep learning system, using a data-centric approach, for detecting, segmenting, and longitudinally tracking brain metastases of any size on pre- and posttreatment MRI	The deep learning system showed high sensitivity and strong generalizability for detecting and segmenting brain metastases, including very small lesions, across internal and external MRI datasets.
Tu J. et al.[31]	China	Oncology	Glioblastoma infiltration detection in peritumoral edema	DL	Detection/segmentation	To develop and evaluate an interactive deep learning framework named the Glioblastoma Infiltrating Area Interactive Detection Framework (GIAIDF) integrating diffusion tensör imaging biomarkers to detect microscopic glioblastoma infiltration within peritumoral edema	The GIAIDF framework can identify microscopic glioblastoma infiltration within peritumoral edema on preoperative MRI and may assist in visualizing infiltrated tumor regions for surgical planning
Tuxunjiang P. et al.[54]	China	Neurology	Stroke severity prediction using multimodal MRI	DL	Prediction/severity estimation	To develop cross-attention vision transformer model using multimodal MRI to predict NIHSS scores and stroke severity	Transformer-based multimodal model accurately predicted stroke severity and could support clinical decision-making
Wang B. et al.[32]	China	Infectious Disease	MRI differentiation of Brucella and tuberculosis spondylitis	DL	Classification/diagnosis	To develop and validate a deep learning-based MRI model for differentiating Brucella spondylitis from tuberculous spondylitis	The proposed model offers promising potential for the diagnosis of BS and TS using conventional MRI. It could serve as an invaluable tool in clinical practice, providing a reliable reference for

							distinguishing between these two diseases
Wang G. et al.[80]	China	Radiology	Carotid artery stenosis detection on non-contrast CT	ML	Classification/diagnosis	To develop and evaluate a radiomics-based machine learning model using non-contrast CT for identifying carotid artery stenosis	Radiomics analysis of non-contrast CT imaging provides an effective, contrast-free means of identifying carotid artery stenosis
Wang H. et al.[81]	China	Neuroradiology/Stroke	Responsible aneurysm identification in SAH patients with multiple aneurysms	ML	Prediction	To develop and test machine learning (ML) models using computed tomography angiography to identify the intracranial aneurysm (IA) responsible for subarachnoid hemorrhage (SAH)	ML models integrating morphological features accurately identified the aneurysm responsible for SAH and outperformed traditional predictive markers
Wang H et al.[56]	China	Radiology	ICH black hole sign identification on CT	ML	Prediction	To develop a self-supervised deep learning framework for automated identification of the black hole sign on head CT to improve prediction of hematoma expansion in intracerebral hemorrhage	The SSL-based deep learning framework accurately identified the black hole sign on head CT and may assist in early prediction of hematoma expansion in intracerebral hemorrhage
Wang K. et al.[33]	China	Orthopedics	Postoperative outcome prediction after tubular microdiscectomy for lumbar disc herniation	DL	Prediction/outcome classification	To evaluate whether dual-plane MRI-based deep learning features combined with clinical features can assess 1-year postoperative outcomes in patients undergoing tubular microdiscectomy for lumbar disc herniation	A model combining MRI-based DL features and clinical features can assess 1-year postoperative outcomes of TMD for LDH

Wang T. et al.[34]	China	Radiology/Stroke	Post-thrombectomy intracranial hemorrhage CT differentiation	DL	Image generation/diagnostic classification	To develop a transformer-based generative adversarial network to generate synthetic material decomposition images from single-energy CT for detection of intracranial hemorrhage after thrombectomy	The proposed trans-GAN improved image generation quality and diagnostic performance, offering a practical single-energy CT-based method for early hemorrhage-versus-contrast differentiation in settings without dual-energy CT
Wang Y. et al.[35]	China	Oncology	Brain metastasis segmentation	DL	Segmentation	To develop and evaluate a Diffusion-CSPAM-U-Net model for the segmentation of brain metastases on CT images and provide a tool where MRI is not accessible	The diffusion-CSPAM-U-Net model showed promising results in segmenting brain metastases on CT
Xia X. et al.[82]	China	Oncology	Glioblastoma versus solitary brain metastasis differentiation	ML	Classification/diagnosis	To develop and validate interpretable machine learning models for differentiating glioblastoma (GB) from solitary brain me-tastasis (SBM) using radiomics features from contrast-enhanced T1-weighted MRI	Machine learning techniques based on radiomics can effectively distinguish GB from SBM, with gradient boosting tree- based models such as LGBMs demonstrating superior performance
Xia X. et al.[69]	China	Neurology	Functional outcome prediction after ICH	ML	Prediction/prognosis	To evaluate ability of peri-hematoma and intra-hematoma radiomic features to predict the 90-day poor functional outcome for spontaneous intracerebral hemorrhage (sICH)	The nomogram integrating clinical-semantic and radiomics signatures accurately predicted 90-day poor functional outcomes for sICH
Xing L. et al.[36]	China	Orthopaedics/Spine	Modic changes detection and grading on lumbar spine MRI	DL	Detection/grading	To develop and evaluate CNN-based models for automated detection and quantitative grading of Modic changes on lumbar spine MRI	The YOLOv8 model showed superior performance compared to YOLOv5 and improved diagnostic consistency, demonstrating potential for assisting

							clinicians in evaluating Modic changes
Xu W. et al.[83]	China	Oncology	Grade 4 glioma molecular subtyping with MRI radiomics	ML	Preoperative molecular subtype classification and prognostic stratification	To develop and validate a multiparametric MRI-based machine learning model for preoperative differentiation of 2021 WHO grade 4 glioma molecular subtypes and to assess its prognostic value	The machine learning model effectively differentiated key grade 4 glioma molecular subtypes and also provided useful survival stratification, supporting noninvasive molecular subtyping before surgery.
Xu X. et al.[84]	China	Neurology	Prediction of cerebrovascular disease related cognitive impairment	ML	Prediction/risk stratification	To establish morphological and radiomic models for early prediction of cognitive impairment associated with cerebrovascular disease (CI-CVD) in an elderly cohort based on cerebral magnetic resonance angiography (MRA)	Radiomics features combined with morphological indicators of cerebral arteries were effective indicators for early signs of CI-CVD in elderly individuals
Yang H. et al.[49]	China	Neurology	Prognostic prediction in acute ischemic stroke after thrombolysis	DL	Prediction/prognosis	To develop and validate predictive models based on diffusion-weighted imaging MRI (DWI-MRI) for assessing the prognosis of patients with acute ischemic stroke (AIS) treated with intravenous thrombolysis	Deep learning models integrating DWI-MRI and clinical features outperformed traditional methods, demonstrating strong generalizability in external validation
Yang Q. et al.[65]	China	Oncology	Pituitary neuroendocrine tumor consistency prediction using mpMRI radiomics	ML	Classification/prediction	To investigate the clinical value of preoperative prediction of pituitary neuroendocrine tumor (PitNET) consistency based on	The mpMRI radiomics model effectively predicted PitNET consistency before surgery and demonstrated differences in predictive performance between 2D and 3D ROI-based models

						multiparametric magnetic resonance imaging (mpMRI) radiomics	
Ye B. et al.[85]	China	Orthopaedics/Spine	Prediction of vertebral artery injury during C2 pedicle screw placement	ML	Risk prediction/classification	To identify risk factors for vertebral artery injury during C2 pedicle screw placement and develop a machine learning model to improve preoperative risk assessment	An ML-based predictive model identified key anatomical risk factors and showed strong performance for estimating vertebral artery injury risk during C2 pedicle screw planning
Yin L. et al.[99]	China	Oncology/Radiology	Preoperative glioma grading using MRI	Hybrid (DL/ML)	Classification/diagnosis	To develop an integrated framework combining radiomics features and deep learning features from multiparametric MRI for accurate preoperative glioma grading	The integrated radiomics-deep learning model significantly improved glioma grading accuracy compared with radiomics only and deep learning only models
Yin S. et al.[100]	China	Oncology	Preoperative glioma grading	Hybrid (DL/ML)	Classification/grading	To develop an integrated deep learning and radiomics framework for accurate preoperative glioma grading using multicenter MRI data	The hybrid radiomics-deep learning model significantly improved glioma grading performance and demonstrated strong diagnostic accuracy across multicenter datasets
Yonar A. et al.[101]	Turkey	Oncology	Brain tumor type classification using MRI	Hybrid (DL/ML)	Classification/diagnosis	To develop swarm intelligence-driven hybrid framework combining deep learning features and optimization algorithms for accurate MRI-based brain tumor classification	The proposed DenseWolf-K framework achieved high classification accuracy with reduced false-negative rates, demonstrating strong potential for automated MRI-based Brain tumor diagnosis

Zahoora U. et al.[37]	Pakistan	Oncology	Brain tumor segmentation on MRI	DL	Segmentation	To develop a federated learning framework with adaptive client selection for accurate brain tumor segmentation	The proposed federated learning model with weak-client elimination improved brain tumor segmentation performance and demonstrated the feasibility of privacy-preserving distributed learning for medical imaging dataset
Zeng L. et al.[53]	China	Neuroradiology	Intracranial aneurysm stability prediction on CTA	DL	Classification/risk prediction	To develop a deep learning based model using CTA images to identify unstable intracranial aneurysms and improve stability assessment	The integrated model combining clinical, morphological, and deep learning features achieved the best performance for predicting aneurysm stability and may assist clinical decision making
Zeng, Q. et al.[86]	China	Oncology	Glioblastoma versus solitary brain metastasis differentiation	ML	Classification/diagnosis	To develop a machine learning radiomics model based on MRI to improve preoperative differentiation between glioblastoma and brain metastasis	The ERSS radiomics based machine learning model demostrated effective diagnostic performance for differentiating GBM from BS and may support clinical decision making
Zhai D. et al.[87]	China	Neuroradiology/Strok e	Hemorrhagic transformation versus contrast extravasation differentiation after mechanical thrombectomy	ML	Classification/diagnosis	To evaluate clinical factors, CT signs, and radiomics features for differentiating hemorrhagic transformation from contrast extravasation in high-density areas after mechanical thrombectomy in acute ischemic stroke	The combined model incorporating radiomics features showed the best diagnostic performance and may assist clinical decision making
Zhao K. et al.[89]	China	Oncology	Differential analysis between PCNSL versus low grade glioma	ML	Classification	To develop and validate an MRI based radiomics model for differentiating early stage atypical primary central nervous system lymphoma from low grade glioma	The MRI based radiomics model demonstrated good diagnostic performance in distinguishing early stage atypical PCNSL from LGG with similar radiological features and may assist in

							early treatment decision making
Zhao K. et al.[88]	China	Oncology	Pituitary adenoma Ki-67 prediction	ML	Prediction	To develop and validate a delta-radiomics machine learning model based on DCE-MRI for preoperative prediction of Ki-67 labeling index in pituitary adenomas	The delta-radiomics model based on DCE-MRI achieved high diagnostic performance for the preoperative assessment of Ki-67 status in pituitary adenomas
Zheng B. et al.[73]	China	Neurosurgery	Cervical spondylotic myelopathy prognosis prediction	ML	Prediction	To develop a radiomics-based machine learning model using 3D cervical spinal cord reconstruction to predict postoperative prognosis in patient with cervical spondylotic myelopathy	The radiomics based machine learning model demonstrated good performance in predicting postoperative prognosis in patients with cervical spondylotic myelopathy and may assist in clinical risk stratification
Zhuang X. et al.[90]	China	Orthopedics	Spine fracture imaging analysis	ML	Classification	To develop and to evaluate radiomics based machine learning models for diagnosing acute thoracolumbar vertebral compression fractures on CT and to assess their impact on radiologist performance	Radiomics based machine learning models demonstrated high diagnostic accuracy for detecting acute vertebral compression fractures and improved diagnostic performance of less experienced radiologists

Abbreviations: AI, artificial intelligence; ML, machine learning; DL, deep learning; DL/ML, hybrid deep learning and machine learning; CNN, convolutional neural network; ViT, vision transformer; LSTM, long short-term memory; GAN, generative adversarial network; SVM, support vector machine; RF, random forest; LASSO, least absolute shrinkage and selection operator; MRI, magnetic resonance imaging; CT, computed tomography; CTA, computed tomography angiography; MRA, magnetic resonance angiography; DWI, diffusion-weighted imaging; fMRI, functional magnetic resonance imaging; DCE-MRI, dynamic contrast-enhanced MRI; mpMRI, multiparametric MRI; HR-VWI, high-resolution vessel wall imaging; NCCT, non-contrast CT; DSC, Dice similarity coefficient; AUC, area under the receiver operating characteristic curve; C-index, concordance index; SSIM, structural similarity index; PSNR, peak signal-to-noise ratio; OS, overall survival; PFS, progression-free survival; mRS, modified Rankin Scale; NIHSS, National Institutes of Health Stroke Scale; IDH, isocitrate dehydrogenase; MGMT, O6-methylguanine-DNA methyltransferase; GTR, gross total resection; EOR, extent of resection; HRQOL, health-related quality of life; DCA, decision curve analysis; PROBAST+AI, Prediction model Risk Of Bias ASsessment Tool for Artificial Intelligence; CLAIM, Checklist for Artificial Intelligence in Medical Imaging; TRIPOD-AI, Transparent Reporting of a multivariable prediction model for Individual Prognosis Or Diagnosis using Artificial Intelligence.
